# Assessment of InSight Landing Site Predictions

**DOI:** 10.1029/2020JE006502

**Published:** 2020-08-07

**Authors:** M. Golombek, D. Kass, N. Williams, N. Warner, I. Daubar, S. Piqueux, C. Charalambous, W. T. Pike

**Affiliations:** ^1^ Jet Propulsion Laboratory California Institute of Technology Pasadena CA USA; ^2^ Department of Geological Sciences State University of New York College at Geneseo Geneseo NY USA; ^3^ Earth, Environmental, and Planetary Sciences Brown University Providence RI USA; ^4^ Department of Electrical and Electronic Engineering Imperial College London London UK

**Keywords:** Mars, landing sites, InSight, remote sensing, surfaces, geomorphology

## Abstract

Comprehensive analysis of remote sensing data used to select the Interior Exploration using Seismic Investigations, Geodesy and Heat Transport (InSight) landing site correctly predicted the atmospheric temperature and pressure profile during entry and descent, the safe landing surface, and the geologic setting of the site. The smooth plains upon which the InSight landing site is located were accurately predicted to be generally similar to the Mars Exploration Rover Spirit landing site with relatively low rock abundance, low slopes, and a moderately dusty surface with a 3–10 m impact fragmented regolith over Hesperian to Early Amazonian basaltic lava flows. The deceleration profile and surface pressure encountered by the spacecraft during entry, descent, and landing compared well (within 1*σ*) of the envelope of modeled temperature profiles and the expected surface pressure. Orbital estimates of thermal inertia are similar to surface radiometer measurements, and materials at the surface are dominated by poorly consolidated sand as expected. Thin coatings of bright atmospheric dust on the surface were as indicated by orbital albedo and dust cover index measurements. Orbital estimates of rock abundance from shadow measurements in high‐resolution images and thermal differencing indicated very low rock abundance and surface counts show 1–4% area covered by rocks. Slopes at 100 to 5 m length scale measured from orbital topographic and radar data correctly indicated a surface comparably smooth and flat as the two smoothest landing sites (Opportunity and Phoenix). Thermal inertia and radar data indicated the surface would be load bearing as found.

## Key Points

- The atmosphere, safe surface, and geologic setting of the landing site were correctly predicted by remote sensing data before landing
- The modeled atmospheric temperature profiles and surface pressure were within 1 sigma of the measured deceleration profile and surface pressure
- InSight’s surface is similar to Spirit’s with low rock abundance, low slopes, moderate dust, and is composed of impact regolith over basalt

## 1 Introduction

Selection of the Discovery program InSight landing site took over 6 years during which engineering constraints were identified and mapped onto Mars, remote sensing data were acquired from Mars Reconnaissance Orbiter, Mars Odyssey, and Mars Express and evaluated, and ~20 sites were progressively downselected, leading to final site certification (Golombek et al., 2017). Engineering constraints important to the selection were elevation (≤ −2.5 km for sufficient atmosphere to slow the descent), latitude (3–5°N for solar power and thermal management of the spacecraft), ellipse size (130 km by 27 km from ballistic entry and descent), and a radar reflective (for altitude determination during descent) and load bearing surface (for stable landing and placement of instruments on the ground). The selected ellipse also met all other landing site constraints with slopes <15° at 84 and 2 m length scales for radar tracking and touchdown stability, low rock abundance (<10%) to avoid impact and spacecraft tip over, constraints to deploy the instruments, which included identical slope and rock abundance constraints at ~1 m scale, and a fragmented fine‐grained regolith ~3–5 m thick for full penetration of the heat flow probe (Golombek et al., 2017). Unlike previous Mars landers and rovers, science objectives (outside of deploying the instruments) did not factor into landing site selection. The final landing site is located in western Elysium Planitia (Golombek et al., 2017).

The InSight lander is a slightly modified rebuild of the Phoenix lander, with three landing legs attached to a central structure that contains electronics and a flat top (or “deck”) holding the science instruments, larger circular solar panels, and an Instrument Deployment Arm (IDA) (Trebi‐Ollennu et al., 2018) to deploy the two instruments, the seismometer (SEIS) (Lognonné et al., 2019) and the Heat Flow and Physical Properties Package (HP^3^) (Spohn et al., 2018) onto the surface. The lander carries two color cameras, the Instrument Context Camera (ICC) and the Instrument Deployment Camera (IDC) inherited from the Mars Exploration Rover and Mars Science Laboratory (MSL) Hazcams and Navcams, whose detectors were replaced with Beyer‐pattern color detectors (Maki et al., 2018). The ICC is a fish eye camera with a 124° by 124° field of view that was attached to the lander just below the deck, providing a wide‐angle view of the workspace where the instruments were deployed. The IDC is a 45° by 45° field of view camera attached to the upper forearm of the IDA (Trebi‐Ollennu et al., 2018). The IDA is 4 degrees of freedom, 1.8 m long arm that moved the IDC to take stereo images with horizontal overlap, take 360° panoramas, image the workspace to select locations to place the instruments, and image under the lander. The arm placed the two instruments within a crescent shaped workspace up to 1.65 m radially from the lander and about 1.5 m to either side. The HP^3^ also carried an infrared radiometer (RAD) mounted under the lander deck on the spacecraft that measured the surface brightness temperatures in two fields of view (“RAD spots”) facing north (opposite of the workspace) (Spohn et al., 2018). These measurements allowed determination of the thermal inertia of surface materials, which have been related to their grain size and/or cementation. Surface data have been used to identify the geologic materials and features present, quantify their areal coverage, determine the basic geologic evolution of the area, and provide ground truth for orbital remote sensing data (Golombek, Grott, et al., 2018; Golombek, Warner, et al., 2020).

The comprehensive evaluation of remote sensing data and entry, descent, and landing simulations conducted during the selection of the landing site gave rise to predictions about the atmosphere and surface (Golombek et al., 2017), which are tested here. If remote sensing data can be related to landing site surfaces, they can be used as ground truth for orbital data (Golombek et al., 1997, 1999, 2005; Golombek, Grant, et al., 2003, 2012), thereby improving the selection of future landing sites and the types of surfaces and materials found on Mars.

## 2 General Predictions

General predictions of surface characteristics made during landing site selection were that western Elysium Planitia would be safe for the InSight landing system and that the instruments could be deployed onto the surface in the workspace of the IDA. Evaluation of remote sensing data from orbit (Golombek et al., 2017; Golombek, Grott, et al., 2018) indicated the smooth plains upon which the InSight landing site is located would be generally similar to the Mars Exploration Rover Spirit landing site (Golombek et al., 2005; Golombek, Crumpler, et al., 2006; Golombek, Grant, et al., 2003) with relatively low rock abundance, low slopes, and a moderately dusty surface. Furthermore, the smooth plains have been interpreted to be composed of an impact fragmented regolith 3–10 m thick underlain by Hesperian to Early Amazonian basaltic lava flows. This is based on geologic mapping, exposures of scarps, rocky ejecta craters (RECs), CRISM spectra, and the presence of wrinkle ridges (Golombek et al., 2017; Golombek, Grott, et al., 2018; Warner et al., 2017; Pan et al., 2020). Observations of the Gusev cratered plains during the Spirit rover traverse (Grant et al., 2004; Golombek, Crumpler, et al., 2006) also indicate an impact generated regolith about 10 m thick that overlies Hesperian basalt flows. Finally, analysis of 100 m scale fresh, RECs in the InSight landing site (Sweeney et al., 2018), and retention ages from crater size‐frequency distributions of craters <1 km (Wilson et al., 2019) shows that the crater degradation rates are consistent with a surface shaped dominantly by impact, mass wasting, and eolian processes (Golombek, Warner, et al., 2020; Grant et al., 2020; Warner et al., 2020), similar to the Gusev cratered plains (Golombek, Crumpler, et al., 2006; Golombek, Grant, et al., 2006). Thus, expectations prior to landing were that the InSight landing site would likely have degraded impact craters that have been filled in to form hollows.

InSight landed safely on 26 November 2018 at 4.502°N, 135.623°E at an elevation of −2,613.426 m (Golombek, Warner, et al., 2020; Parker et al., 2019) with respect to the Mars Orbiter Laser Altimeter (MOLA) geoid (Smith et al., 2001). Images acquired by the High‐Resolution Imaging Science Experiment (HiRISE) (McEwen et al., 2007) based on the initial radio tracking location show that the lander touched down in the northwest central portion of the landing ellipse in western Elysium Planitia (Golombek, Warner, et al., 2020; Parker et al., 2019). The instruments were successfully deployed onto the surface several months later.

Since landing, a large number of stereo color surface images, mosaics, and panoramas from the IDC have been acquired (Golombek, Warner, et al., 2020). To place the instruments on the surface, mosaics of the instrument deployment workspace immediately south of the lander at 0.5–2 mm/pixel were taken. Three complete panoramas during the morning, afternoon, and evening were also acquired along with images of the lander, terrain beneath the lander, its footpads, and the RAD spots. These surface images provide information on the characteristics of the landing site that can be compared with orbital measurements.

**Figure 1:**
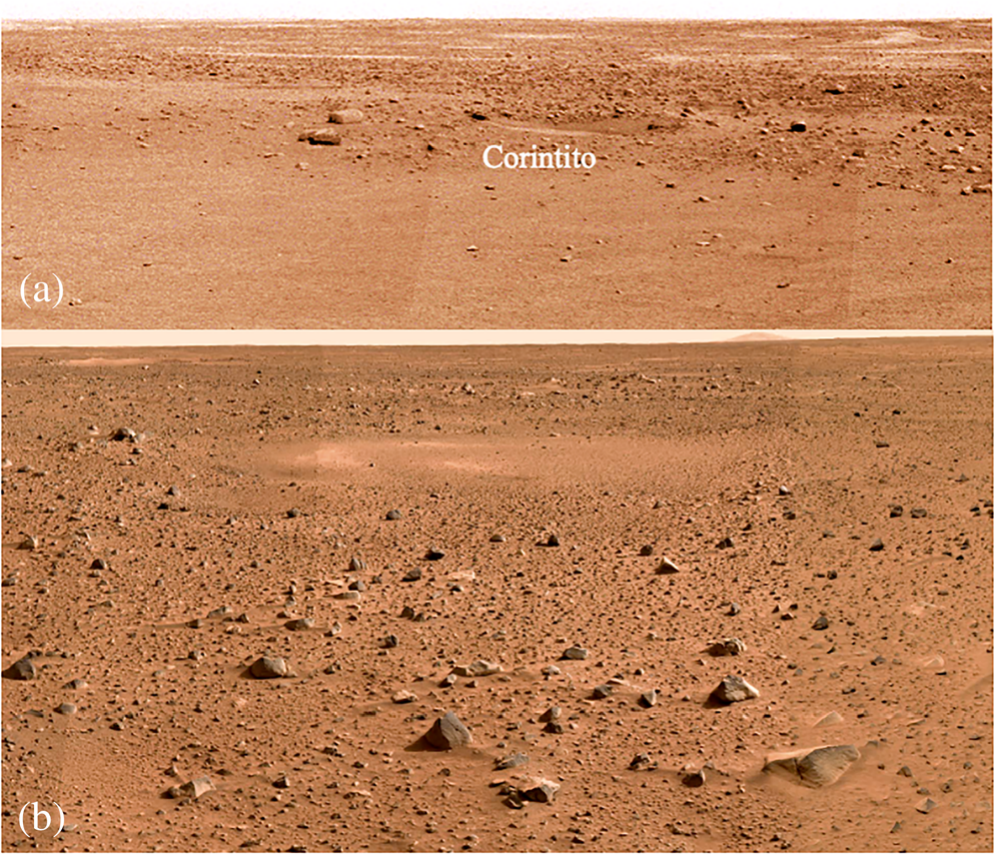
Surface mosaics of the InSight landing site (a, toward the southeast) and Spirit landing site (b, toward the southwest) showing their general similarity. Note that both sites have relatively low rock abundance, low slopes, and moderately dusty surfaces that have been dominantly shaped by impact, mass wasting, and eolian processes. Note exceptionally smooth *Homestead hollow* surface at InSight with few rocks in the foreground and soil filled hollows (degraded, filled in craters) in both scenes. Corinto secondary crater, *Corintito* (3 m diameter) is about 20 m away from the InSight lander at the edge of *Homestead hollow*. The InSight panorama was acquired on Sol 14. The Spirit panorama was acquired on Sols 6–10 as part of the mission success pan.

The lander is located within a quasi‐circular depression, interpreted to be a degraded ~27 m diameter impact crater, informally named *Homestead hollow* (Figure 1), with a smooth sandy granule‐ and pebble‐rich surface adjacent to a slightly rockier and rougher terrain (Golombek, Warner, et al., 2020). About ten 1–10 m diameter impact craters can be seen within 20 m of the lander (Grant et al., 2020; Warner et al., 2020). Some of these craters have very little relief and are filled with fine‐grained material. In contrast to the smooth surface of *Homestead hollow*, the surface to the west of the lander that extends into the distance at most azimuths away from the lander is rougher and rockier with more centimeters to tens of centimeters size rocks. Many of the larger rocks closest to the lander have a dark gray color and appear very fine grained, consistent with aphanitic, dark mafic rocks (basalts) as expected. A fuller description of the geology of the landing site can be found in Golombek, Warner, et al. (2020) and companion papers in this issue (e.g., Grant et al., 2020; Warner et al., 2020; Weitz et al., 2020).

All of the predictions of the general physical characteristics of the surface appear correct as determined by investigations based on data from the lander (Figure 1). At the broadest level, the surface appears similar to the Spirit landing site with relatively low rock abundance, low slopes, and a moderately dusty surface. In addition, we have compared the specific remote sensing data at the landing site to the surface characteristics observed by the lander.

## 3 Atmosphere

The state of the atmosphere is a strong factor in successfully landing on Mars. There must be sufficient atmosphere for the lander to decelerate and reconfigure itself for touchdown before reaching the surface. In addition, atmospheric variations on short length scales can drive oscillations and resonances that can cause the landing system to exceed its design envelope (Grover et al., 2011). To make sure the landing system is able to perform properly over the range of possible atmospheric conditions, atmosphere models were prepared for Monte Carlo testing of the landing system (e.g., Desai et al., 2006; Prince et al., 2011). In addition, to avoid late surprises, the atmosphere was monitored in the period prior to landing to confirm that current conditions match the expectations and atmospheric models used for design.

The InSight landing season at *Ls* = 295° (determined by the launch opportunity and orbital mechanics) is particularly difficult in terms of the range of potential atmospheric conditions (Golombek et al., 2017). It is shortly after the summer solstice and perihelion, when Mars is closest to the Sun and thus receiving maximum insolation. Southern spring and summer are known as the dusty season (Kahre et al., 2017). During this period, not only can small local dust storms occur but larger regional and the largest global dust events (GDEs) also occur. Large regional events can modify the temperatures, and thus atmospheric density profile, across broad regions of Mars, and the rare GDE can drastically modify it everywhere. Large regional storms have not occurred at the landing season in recent years (Kass et al., 2016); however, there is some evidence for their occurrence at *Ls* ~ 295° from telescopic observations (Martin & Zurek, 1993; McKim, 1999). Multiple GDEs have occurred during the InSight landing season in the past (Shirley et al., 2019; Zurek & Martin, 1993). Four primary atmospheric models were developed to accommodate the range in possible conditions when the spacecraft arrived (Golombek et al., 2017). These models are the background, representing the seasonal atmospheric behavior at times without large regional or global dust storms; a large regional dust storm; a global dust storm; and a decaying dust storm. These four models resulted in significantly different performances and landing locations relative to the entry point at the top of the atmosphere. Changes in the entry target, by trajectory correction maneuvers prior to landing day, and changes to the parameters controlling the descent significantly improved the performance (and reduced the risk of failure) for the atmosphere that was predicted on landing.

The Martian weather during the dusty season in 2018 was eventful. In early June, a GDE started (Guzewich et al., 2019; Kass et al., 2020). The storm began early in the dusty season at *Ls* = 186° and evolved to eventually encompass the entire planet. The Curiosity rover, in Gale crater only ~600 km away from the InSight landing site, measured a significant enhancement of dust (Guzewich et al., 2019), and column opacities above Curiosity and Opportunity reached values in excess of 8 and 10.8, respectively (among the highest measured from the surface). The very early seasonal start to the 2018 GDE was favorable for InSight. The landing season was beyond the longest known duration for a GDE and in particular it was later than the end of the 2001 GDE (Smith et al., 2002) that started at almost the same season. However, the early start and expected end before landing did not eliminate the risk. In 1977, the Viking mission observed two GDEs in the same dusty season. One that started early and one that started around the solstice (Zurek & Martin, 1993) and reached its peak atmospheric impact right at the InSight landing season.

As expected, the GDE ended by early November, prior to the InSight landing (Guzewich et al., 2019; Kass et al., 2020). At this time, four months after the event reached its mature phase, all of the enhanced dust had sedimented back onto the surface. Atmospheric dust loads had returned to seasonally expected background conditions, as had global atmospheric temperatures. All were within the range of values seen in previous Mars years without a GDE, albeit conditions were among the dustier cases for previous years at the landing season. The lack of atmospheric “memory” of the GDE was expected due to the very short radiative timescale for the thin Martian atmosphere (Wolff et al., 2017).

Global observations of the atmosphere by Mars Climate Sounder (McCleese et al., 2007) and Mars Color Imager (Malin et al., 2001) on Mars Reconnaissance Orbiter (Zurek & Smrekar, 2007) and surface observations by Rover Environmental Monitoring Station (REMS) (Gómez‐Elvira et al., 2012) on Curiosity started in early November. This was delayed to wait for the GDE to finish clearing. (Otherwise, observations would not match the expected conditions on landing day.) Monitoring continued through to landing day. Fortunately, no additional large dust events occurred during this timeframe. Conditions were very close to the background conditions predicted during development and remained near the climatological average. There was some modest day‐to‐day variability or weather. The observations continued to landing day to allow the comparison of the remote sensing and REMS data with measurements derived from the InSight entry and surface observations.

There are no instruments on InSight to directly measure the atmosphere during entry and descent. However, the deceleration profile was used to reconstruct the density (temperature and pressure) profile as has been done on previous missions (Blanchard & Desai, 2011; Chen et al., 2014; Magalhaes et al., 1999; Withers & Smith, 2006). The reconstruction (Karlgaard et al., 2020) compared well to the climatological background as well as the atmosphere models. The models do not show any systematic bias in the temperature profile, although as expected the reconstructed profile has higher frequency vertical structure not captured in the model ensemble (some of it is captured in individual profiles). The reconstructed temperature profile generally lies within the 1*σ* variability envelope of the models. It is always within the 3*σ* variability envelope, except for the lowest few kilometers of the reconstructed profile which has very large uncertainties (that encompass the model profiles).

Although InSight has a pressure sensor (Banfield et al., 2019), it did not record data during landing or for several sols after landing (Banfield et al., 2020). The initial relevant pressure observations are influenced by the seasonal pressure trend (Smith et al., 2017). Using a linear extrapolation from data over Sols 5 through 40 (primarily Sols 14 through 40) at the landing local mean solar time results in an outstanding match to the REMS climatological and landing day data. All of the models are within 0.3%, and some are within 0.1% of the extrapolation. The 1*σ* uncertainty in the extrapolation is 0.3%, and there is also a day‐to‐day variability of up to 0.6% due to weather in the InSight data. The model surface pressure variability was 4.4% (1*σ*) to account for the uncertainty, the extrapolation from Curiosity to InSight, and weather.

Despite the close correspondence between the model and reconstructed temperature profiles as well as the surface pressure, the reconstructed density profile is consistently around 1*σ* below the mean model density. It is within the 2*σ* envelope for both the climatological and approach models at all altitudes. Furthermore, there are individual profiles from the model that are a much closer match to the reconstructed profile. The difference between the model and the actual atmosphere leading to the density difference is most likely in the lowest ~7 km, below the lowest point in the reconstructed profile (where the parachute deployed). The atmosphere encountered by InSight was well within the capabilities of the landing system as evidenced by the successful landing, within the desired landing region (Golombek, Warner, et al., 2020), and within the expected model conditions for the background atmosphere.

## 4 Thermal Inertia and Albedo

Thermal inertia is a measure of how rapidly (or slowly) materials change temperature and is related to material properties such as bulk density, particle size, and cementation. On Mars, loose, very fine grained dust changes temperature rapidly during the diurnal cycle and so has low thermal inertia (and high albedo). In contrast, rocks or duricrust (cemented soil‐like materials) change temperature more slowly and have higher thermal inertia (Christensen & Moore, 1992; Mellon et al., 2008). Large portions of Tharsis, Elysium, and Arabia Terra have very low thermal inertia and high albedo indicating surfaces that are dominated by fine‐grained bright dust that could be meters or more thick (Christensen & Moore, 1992). Such areas have been eliminated as possible landing sites for all previous spacecraft (Golombek et al., 2017; Golombek, Grant, et al., 2003, 2012) due to concerns about dust coating the solar panels and reducing power, as well as sinkage, as meters‐thick dust is neither load bearing nor trafficable (Golombek, Haldemann, et al., 2008). All these missions (including InSight) have had requirements that the thermal inertia be greater than 100–140 J m^−2^ K^−1^ s^−1/2^, to ensure a radar reflective and load bearing surface.

The InSight landing site is generally similar in global thermophysical properties to the Viking, Spirit, Phoenix, and Curiosity landing sites with moderate thermal inertia and intermediate to relatively high albedo. All of these sites fall into Unit C of the three global thermal inertia‐albedo units (or modes) that make up ~80% of Mars (Putzig et al., 2005; Putzig & Mellon, 2007). The thermal inertia and albedo of the InSight landing site is most similar to the Viking Lander 2 (VL2) site, which has slightly higher albedo and slightly lower thermal inertia than the Viking Lander 1, Spirit, and Curiosity landing sites (Golombek et al., 2017) that has been attributed to more drift (dust) deposits at VL2 (Golombek, Haldemann, et al., 2008).

**Figure 2:**
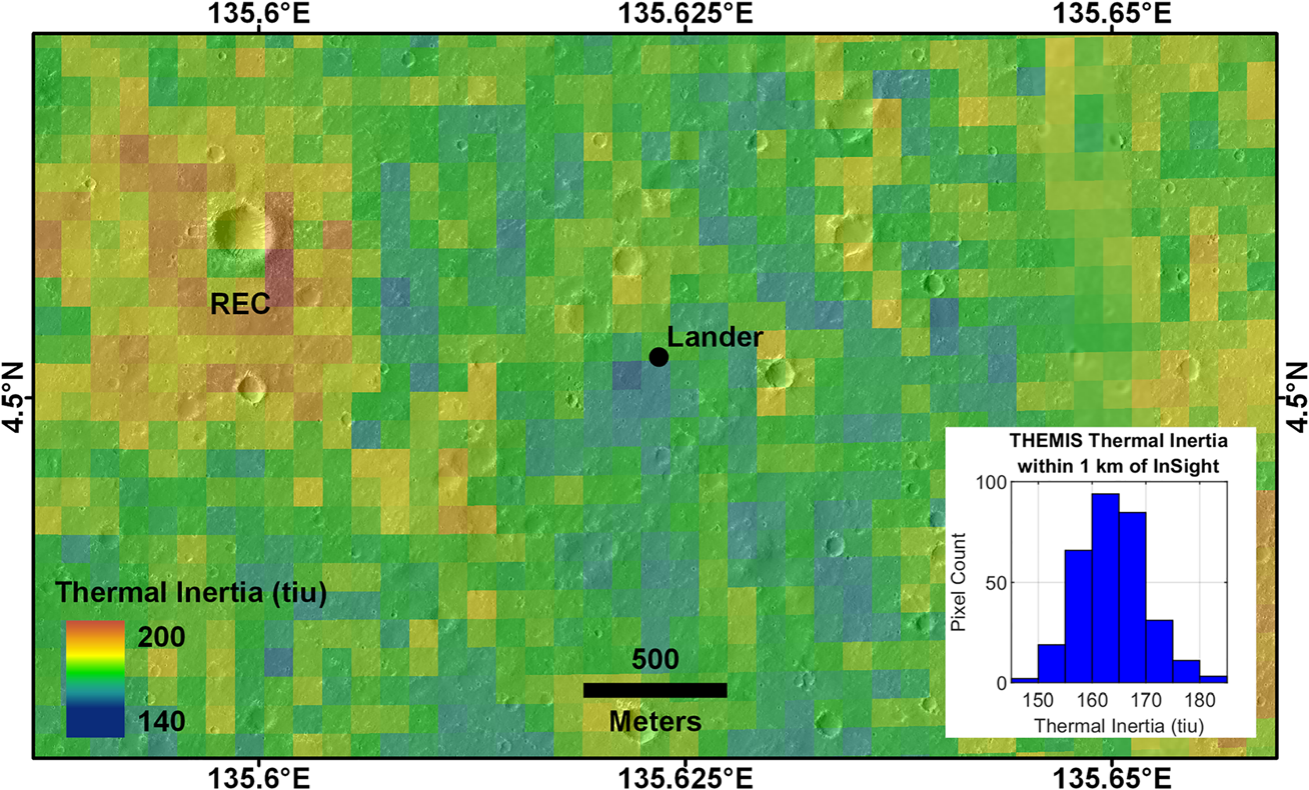
Thermal inertia map of InSight from predawn temperature data acquired by THEMIS (100 m spatial scale) between MY 30 and 32 during low‐dust seasons to minimize the atmospheric impact on the derived values that was created during landing site selection (Golombek et al., 2017). Note the limited range of thermal inertias across the site (145–185 J m^−2^ K^−1^ s^−1/2^ or thermal inertia units, tiu), with a mean of ~164 J m^−2^ K^−1^ s^−1/2^ within 1 km of the lander, indicating fairly homogeneous physical properties. The lander is located in a pixel with a thermal inertia of 166 J m^−2^ K^−1^ s^−1/2^. Note higher thermal inertia associated with the rocky ejecta crater (REC).

Orbital Thermal Emission Spectrometer (TES, 3 km/pixel resolution) nighttime thermal inertia (Putzig & Mellon, 2007) of the landing site is ~230 J m^−2^ K^−1^ s^−1/2^ (average of a 236 J m^−2^ K^−1^ s^−1/2^ that the lander is in and the adjacent pixel 130 m to the south). Thermal Emission Imaging System (THEMIS, 100 m/pixel) thermal inertia (Golombek et al., 2017) is 166 J m^−2^ K^−1^ s^−1/2^. Both are about average for the landing site. Figure 2 shows the THEMIS thermal inertia map at 100 m/pixel of the region around the lander (Golombek et al., 2017). The pixel containing the lander is near the median of the thermal inertia, which increases around the RECs in accordance with their higher rock abundance (Golombek et al., 2017).

The relatively low thermal inertia of the InSight landing site (~200 J m^−2^ K^−1^ s^−1/2^) was interpreted to be due to surface materials composed predominantly of unconsolidated sand size particles or a mixture of slightly consolidated or cohesive soils (cohesions of less than a few kilopascals), some rocks, and thermally thin coatings of dust (e.g., Golombek et al., 2017). The lack of pronounced seasonal variations in the THEMIS‐derived thermophysical properties suggests an absence of a steep inertia contrast within the top few tens of centimeters (Golombek et al., 2017). The relatively high albedo of the InSight landing site: 0.24, TES at 7.5 km/pixel (Christensen et al., 2001) and 0.25, infrared thermal mapper (IRTM) at 60 km/pixel (Pleskot & Miner, 1981) argues for a thin coating of dust similar to the VL2 landing site and dusty portions of the Gusev cratered plains. The InSight and VL2 landing sites also both have dust cover indices (DCI) of 0.94, due to thin surface coatings of dust (Ruff & Christensen, 2002).

These interpretations from orbital remote sensing data are all consistent with observations made from the surface. The HP^3^ RAD diurnal temperature measurements yield an average thermal inertia of ~200 J m^−2^ K^−1^ s^−1/2^ (Mueller et al., 2019; Golombek, Warner, et al., 2020), which is very similar to the higher‐resolution orbital measurements (TES and THEMIS). This thermal inertia corresponds to an unconsolidated particle size of fine sand (~150 μm). Pebble and rock counts in the RAD spots and on the surface surrounding the lander (section 5) show little area (<2%) covered by rocks large enough (>3 cm diameter) to noticeably increase the thermal inertia (Golombek, Haldemann, et al., 2003), indicating that the surface is dominated by sand size particles. Although duricrust has been observed in pits beneath the lander, any cementation does not appear to significantly influence the bulk thermal inertia and the duricrust is only thought to be ~10 cm thick (Golombek, Warner, et al., 2020; Hudson et al., 2020). A shallow subsurface of predominantly unconsolidated fine‐grained material is also consistent with the low seismic velocities measured by SEIS (Lognonné et al., 2020).

**Figure 3:**
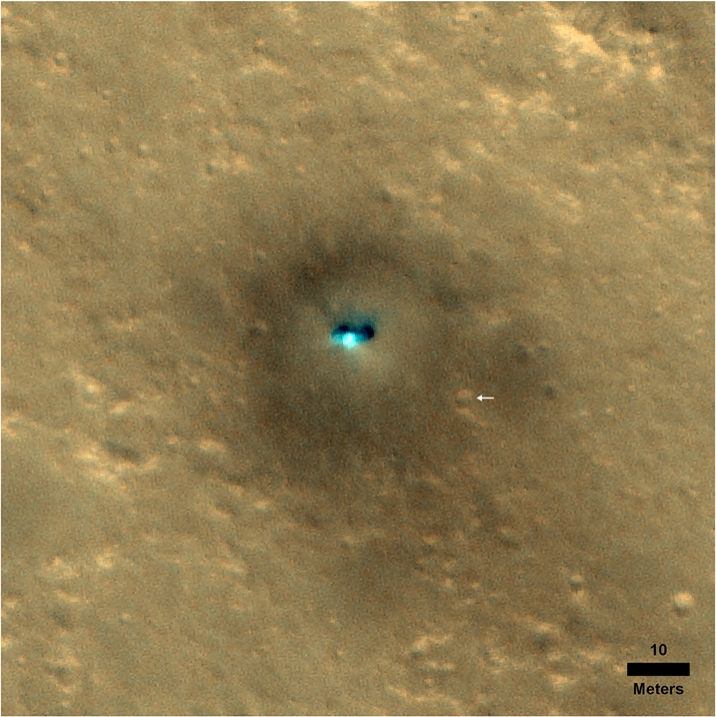
HiRISE image acquired on 6 December 2018 showing the InSight lander and the ~20 m radius low‐albedo spot around the lander. The areal extent of the spot and ~35% reduction in albedo relative to the surroundings can be explained by the removal of a thin coating of surface dust similar to other landers. White arrow points to *Corintito* crater shown in Figure 1. Portion of HiRISE image ESP_057939_1845_RGB NO MAP. North is up, and image has been stretched for contrast.

**Figure 4:**
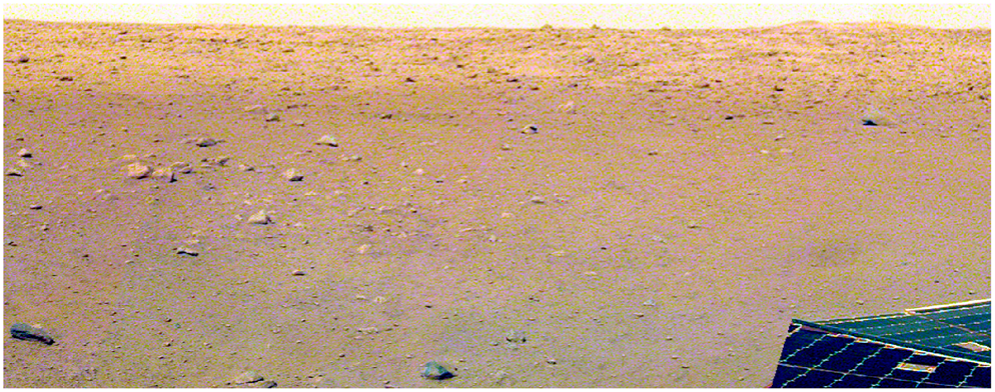
Portion of color surface panorama to the north of the InSight lander acquired on Sol 14. The darker surface where dust has been removed extends out to 20 m from the lander, with brighter, dustier surface beyond. Note the rockier surface to the west, smooth plains to the east to the rim of *Homestead hollow*, and three rocks (*The Pinnacles*) and eolian bedform (*Dusty Ridge*) on the horizon.

The expectation of a moderately dusty surface similar to the dusty portion of the Gusev cratered plains is consistent with the reddish color of the landscape (Golombek, Warner, et al., 2020) and the extensive dark spot produced by the landing thrusters that removed the dust (Williams et al., 2019) (see also section 10). A large, low albedo spot centered on the lander is present in HiRISE images taken about a week after landing (Golombek, Warner, et al., 2020) (Figure 3). The dark spot reaches north about 20 m (Figure 4). Using the method of Daubar et al. (2016), it has ~35% lower relative albedo, compared to a background sample in HiRISE RED images before and after landing (Golombek, Warner, et al., 2020). Note that the albedo derived from fitting the RAD diurnal curves to derive the thermal inertia is 0.15–0.16, which is also 35% lower than the TES albedo of 0.24. The reduction in relative albedo and the size of the dark spot is not unlike those observed around Phoenix and some of the hardware from the Mars Science Lander spacecraft (Daubar et al., 2015). Those sites also both have broadly similar regional prelanding albedos (~0.20–0.24; Golombek et al., 2017). The prelanding dust cover index (DCI) of the InSight landing site (0.94; Golombek et al., 2017) indicates a surface with a thin coating of dust. For comparison, MSL has a similar DCI of 0.95. This indicates that the dark spot resulted from disturbance or removal of that dust from the retrorockets used during the descent and landing of the spacecraft, similar to previous landers.

## 5 Rock Abundance

**Figure 5:**
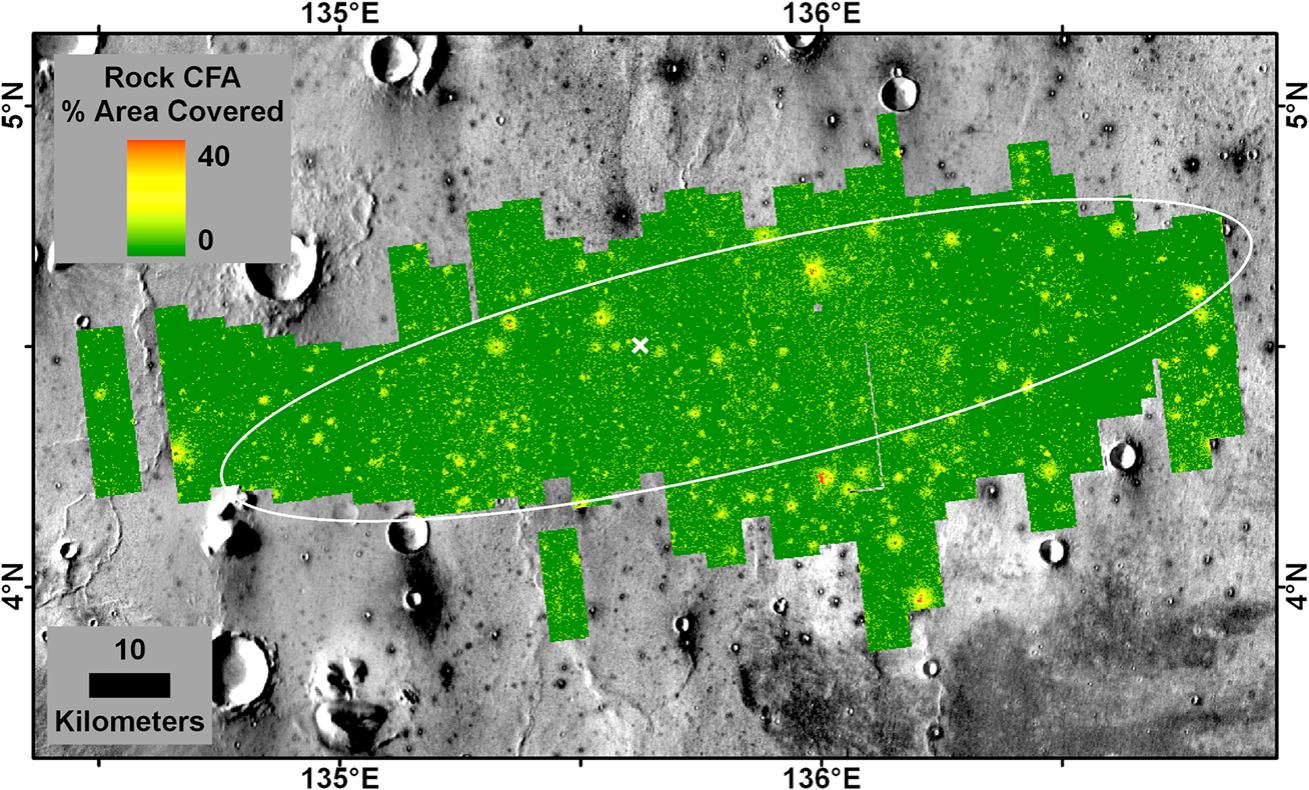
The cumulative fractional area covered by all rocks (0–40%) from the fit of rocks between 1.5 and 2.25 m diameter (or rock abundance) in HiRISE images to the exponential model in 150 m tiles for the InSight landing ellipse. Rock abundance is concentrated around sparse rocky ejecta craters with most smooth terrain having zero rock abundance. The average cumulative fractional area covered by rocks of these 150 m tiles is 1.2%. Note that InSight landed (white X) in a rocky portion of the ellipse. Rock measurements are from Golombek et al. (2017). Background is the THEMIS daytime mosaic in which dark halo craters have higher thermal inertia from rocky ejecta.

Orbital estimates of rock abundance in the landing ellipse indicated a surface with a very low average rock abundance (Golombek et al., 2017). Measurements of rocks in HiRISE images utilizing the rock shadow segmentation, analysis, and modeling method that was successfully developed for the Phoenix landing site selection (Golombek, Huertas, et al., 2008) and improved upon for the MSL landing site selection (Golombek, Huertas, et al., 2012) shows that rocks are concentrated around sparse RECs (Golombek et al., 2017). Counts of rocks and their size‐frequency distributions in 150 m^2^ areas fit to exponential models of the cumulative fractional area (CFA) of rocks versus diameter on Mars (e.g., Golombek, Huertas, et al., 2008, 2012) show the CFA varies from around 35% adjacent to RECs to ~1% in smooth plains away from RECs (Golombek et al., 2017) (Figure 5). Average rock abundance (or CFA) of the 150 m by 150 m square tiles across the ellipse is 1.2%; a single size‐frequency distribution of all of the rocks within the ellipse yields a rock abundance of around 6% and is generally comparable with rock distributions measured at the Phoenix and Spirit landing sites (Golombek et al., 2017). These low rock abundances are also consistent with thermal differencing estimates of rock abundance (<5%) from IRTM and TES data (Christensen, 1986; Nowicki & Christensen, 2007).

**Figure 6:**
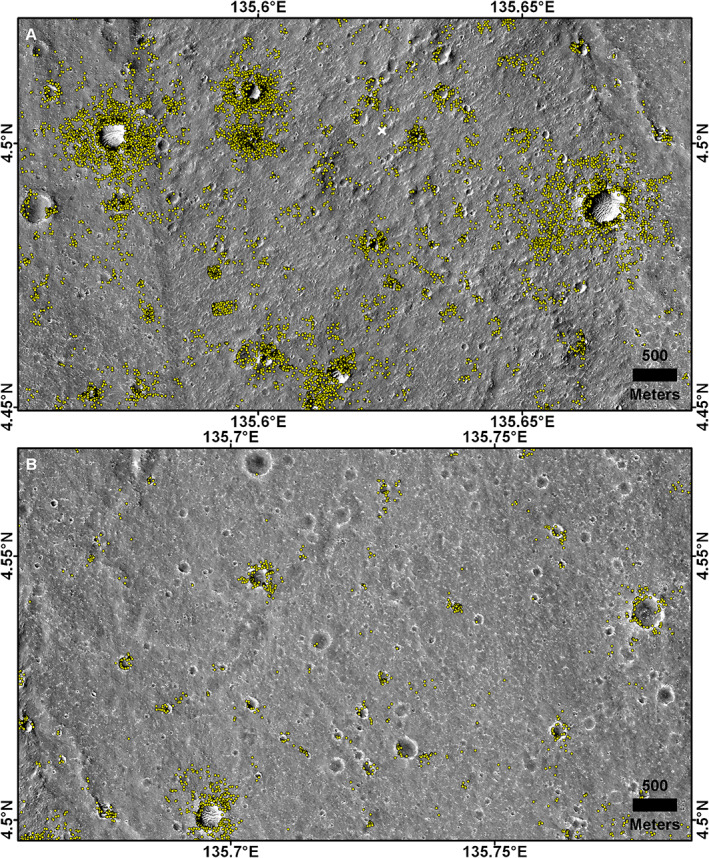
Individual rocks (mostly >1 m) detected (yellow dots) using the semiautomated machine vision shadow technique in sharpened HiRISE images shown used to create the rock abundance map (Golombek et al., 2017) (Figure 5). (a) is area nearby the lander, and (b) is about 8 km to the east characterized by more smooth terrain and fewer rocks. Note the rocks are dominantly surrounding rocky ejecta craters (RECs), with the closest RECs to the lander located about 400 m to the east‐southeast of the lander. Also note the relatively high concentration of rocks nearby the lander that are not associated with RECs. The top half (a) includes parts of images ESP_036761_1845 (center), ESP_039135_1845 (left), and ESP_032014_1985 (right). The bottom half (b) includes ESP_040203_1845 in all but the bottom left corner, which is covered by ESP_032014_1985.

InSight landed in a rockier than average portion of the ellipse with a number of large RECs nearby (Figure 5). The percentage of rocky versus total number of fresh craters 30–60 m diameter is 60–70% within the 5 km^2^ that includes the lander (Golombek, Grott, et al., 2018). In addition, there are five RECs within 3 km of the lander that produce a higher than average background rock abundance on the plains. This is illustrated in Figure 6, which shows all the HiRISE detected rocks measured within a 5 km box near the lander versus those detected about 8 km to the east of the lander. Within the 150 m^2^ area that contains the lander in which rocks were counted in HiRISE, no rocks larger than 1.5 m diameter were measured, which was interpreted as 0% rock abundance (6% CFA in the landing simulations) (Golombek et al., 2017). The closest HiRISE detected rock >1.5 m diameter is 250 m away from the lander.

**Figure 7:**
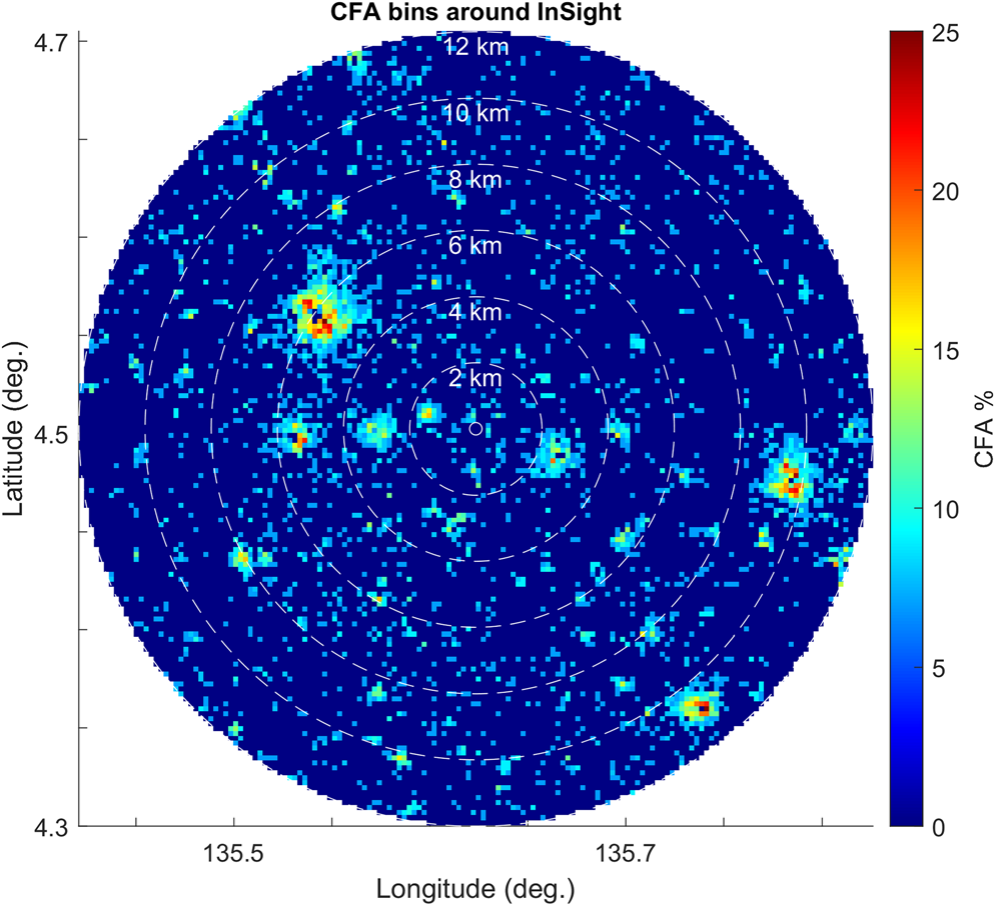
Map of the CFA as determined in 150 m square tiles from measurements of shadows in HiRISE images (with the lander at the center). The immediate area around the lander (within 0.5 km) has few detected rocks. Rocks farther away are concentrated around rocky ejecta craters. Rock abundances are from Golombek et al. (2017).

**Figure 8:**
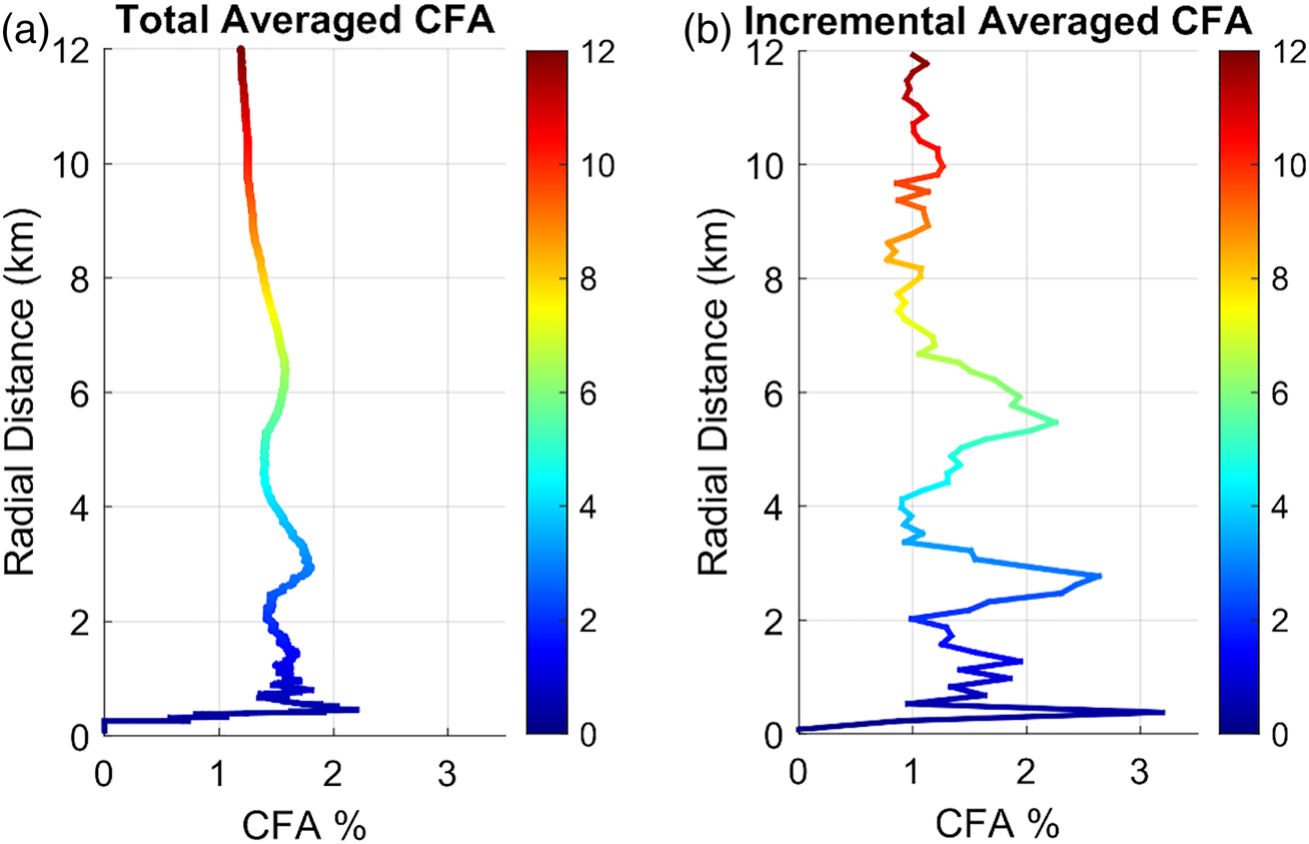
Graphs of the CFA shown in Figure 7 with radial distance from the lander. Graph (a) shows the cumulative average rock abundance with distance calculated by taking the average of all of the 150 m CFA measurements within the distance shown. Graph (b) shows the incremental average rock abundance in 150 m annuli calculated by taking the average of all 150 m CFA measurements at 150 m annuli at the distance shown. Rock measurements are from Golombek et al. (2017).

To further evaluate how rock abundance varies around the lander, Figure 7 shows the CFA in 150 m counting areas with radial distance out to 12 km. The lander is located in an area with no detected rocks and has very few rocks within a few hundred meters. About 4–5 large RECs are found between 1 and 2 km from the lander, about three are found about 3 km away, and about three are found about 6 km away. Graphs of CFA in 150 m tiles measured radially (Figure 8) show that CFA is very low within a few hundred meters from the lander and then peaks at distances with large RECs at about 0.5 km (Figure 6a), 3 km, and 5.5 km before becoming close to the average rock abundance for the ellipse (1.2%) beyond 8 km. These plots show that the lander is located in a 7 km radius area that is rockier (1.5–2%) than the average (1.2%) for the ellipse, but within a few hundred meter radius is less rocky than the average.

**Figure 9:**
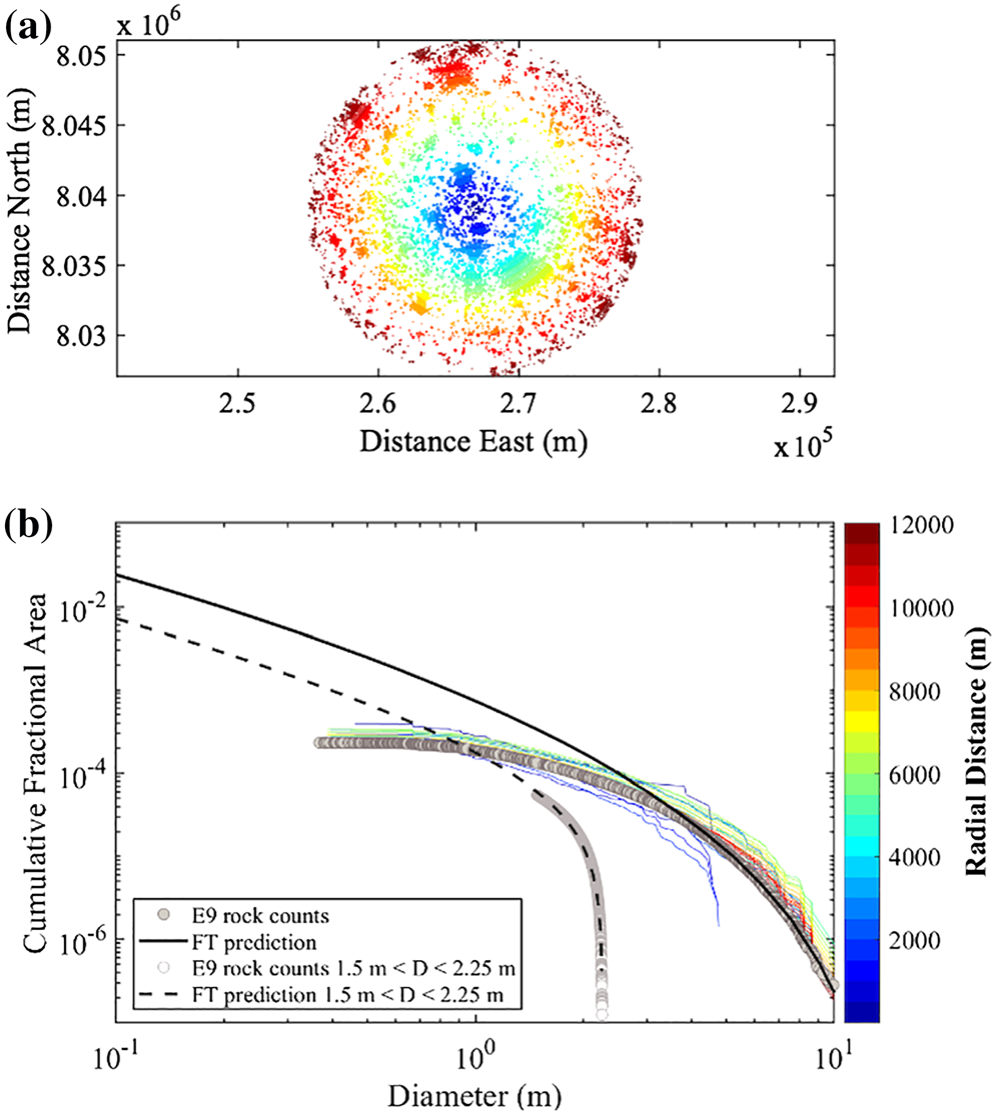
Plot of rocks color coded by radial distance in 500 m steps from the lander (a) and the corresponding size‐frequency distribution of CFA versus diameter (b). Rock shadow detections of all diameters (gray circles in b) as well as those between 1.5 and 2.25 m over 35 complete HiRISE images in the landing ellipse are from Golombek et al. (2017). The solid black and black dashed lines in (b) are the predictions from fragmentation theory for all diameters and those between 1.5 and 2.25 m prior to landing, respectively (Golombek et al., 2017). The blue lines indicate distributions within a 4 km radius, yellow around 7 km radius, and red beyond 9 km radius from the lander (in b). The fragmentation predictions for the CFA and slope of the size‐frequency distribution matches measurements from the lander for diameters <0.05 m shown in Figure 10.

The size‐frequency distributions of rocks binned by distance from the lander (not in 150 m tiles) show a similar pattern (Figure 9). The size‐frequency distribution versus diameter plots for areas within 2 km of the lander falls below the average produced from all rocks in the landing ellipse with fewer detections and a steeper distribution above 4 m diameter. At 3–7 km distances, the size‐frequency distributions versus diameter plots generally exceed the average and the slope of the distribution is similar to the average for diameters larger than 4 m. This increase is consistent with the inclusion of large RECs at these distances. These results are consistent with the results based on the CFA in 150 m square tiles.

Average rock abundances of <2.5% (CFA) in 150 m square tiles nearby the lander are consistent with thermal differencing estimates for the location of the lander (which are averages over larger pixel areas). The IRTM rock abundance (Christensen, 1986) is 4% (~60 km pixel), and the nearest TES rock abundance, about 10 km to the east, is 3.3% (7.5 km pixel) (Nowicki & Christensen, 2007). The average TES rock abundance within 20 km of the lander is 3.7% (11 pixels).

**Figure 10:**
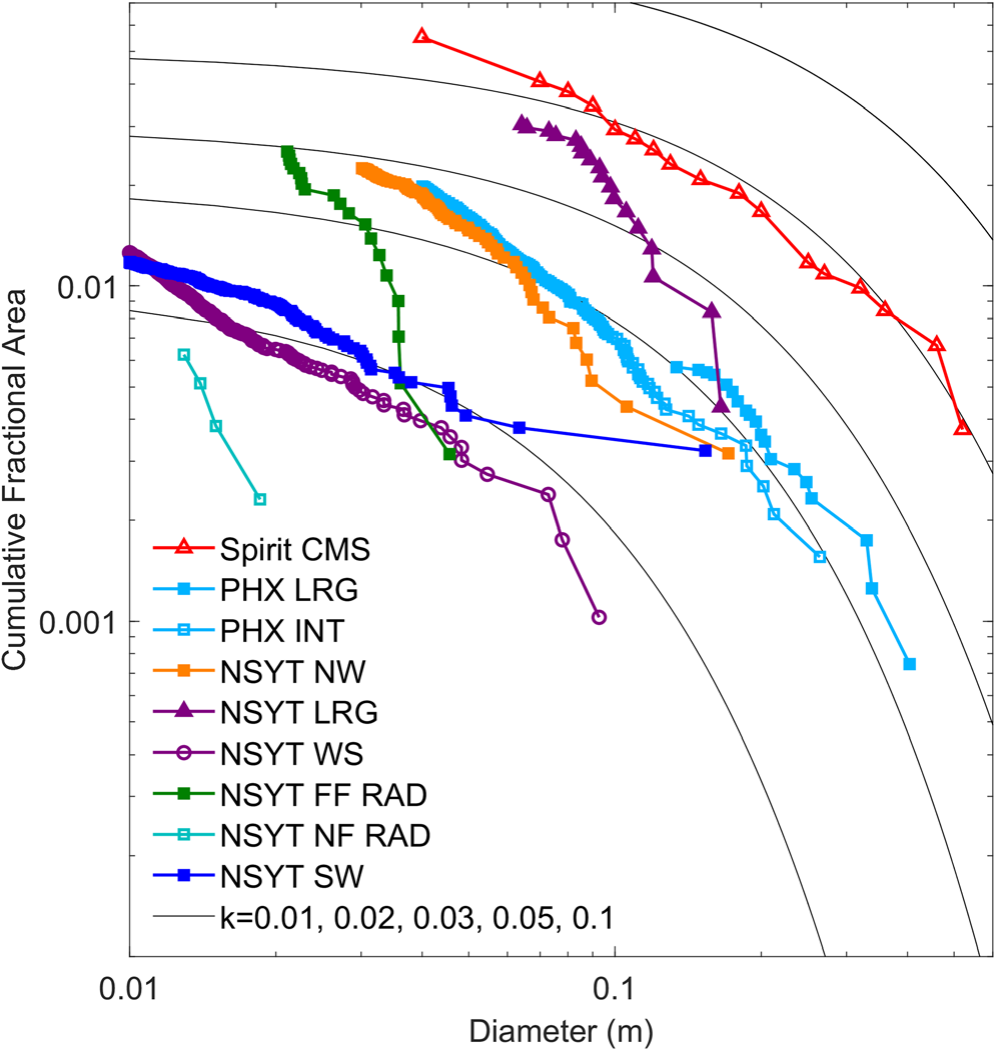
Cumulative fractional area versus diameter of rocks near the InSight lander and the Spirit (Spirit CMS for Columbia Memorial Station) and Phoenix (PHX) landing sites. Exponential model size‐frequency distributions are shown for rock abundances (k) of 1%, 2%, 3%, 5%, and 10% (Golombek & Rapp, 1997). Rocks smaller than 10 cm diameter in the workspace fall just below the 1% model to about 2 cm diameter, where the distribution steepens and just exceeds 1% rock abundance at 1 cm. The size‐frequency distribution of rocks to the southwest are similar for diameters <7 cm, with one rock >10 cm near the 2% model. Rocks with diameters of 20–7 cm to the northwest fall just below the 2% model; the distribution rises to ~3% rock abundance at 2 cm diameter and is similar to the rock size‐frequency distribution at the Phoenix landing site. The largest rocks with diameters of >10 cm toward the south fall between the 2% and 3% model curves. At smaller diameters the rock distribution is similar to the Spirit landing site (~4%). Taken together, the smooth terrain near the lander has a rock abundance of 1–2% and the rockier terrain has a rock abundance of 2–4%. Surface rock counts are the area to the northwest (NSYT NW) and southwest (NSYT SW), the area to the south with the largest rocks (NSYT LRG), the workspace (NSYT WS), and the near and far RAD spots (NSYT NF RAD and NSYT FF RAD, respectively). Spirit CMS rocks are from Golombek, Crumpler, et al. (2006), Phoenix largest rocks (PHX LRG) are from Golombek, Huertas, et al. (2012), and Phoenix intermediate area (PHX INT) is from Heet et al. (2009). All InSight counts are described in Golombek, Warner, et al. (2020), except for NYST SW, which consists of 271 rocks identified over 5.74 m^2^ ranging in diameter from 0.4 to 15.4 cm measured in D019L0119_607101993EDR_F0103_0100M3.

Measurements of rocks in lander images indicate low rock abundance (Figure 10) that is consistent with orbital estimates. Rock counts were made in areas around the lander that include the lowest abundance (the smooth plains in the instrument deployment workspace to the south), the highest abundance (5 m south in the rocky terrain), and those in between (rocky terrain northwest and southwest of the lander and the RAD spots) (Golombek, Warner, et al., 2020). For rocks 6–20 cm diameter, the CFA versus diameter size‐frequency distributions (Figure 10) is comparable to exponential rock size‐frequency models for 1–4% rock abundance, which have been used to model rock populations for landing sites on Mars (Golombek & Rapp, 1997; Golombek, Haldemann, et al., 2003; Golombek, Huertas, et al., 2008, 2012). Rocks cover 1–3% of the area, and the distributions are steeper than the exponential models for diameters smaller than 4 cm. Only ~2% of the surface in the far RAD (and 0% in the near) spot are covered by rocks >3 cm, which cover too little area and are too small to have any effect on the thermal inertia (Golombek, Haldemann, et al., 2003). The rock distributions around the lander are similar to those measured at the Phoenix (2%) and Spirit (4%) landing sites (Golombek, Crumpler, et al., 2006; Golombek, Huertas, et al., 2012) and are consistent with orbital estimates prior to landing. For rocks less than 4 cm diameter, the steep increase in the size‐frequency distributions most closely resembles the pebble‐rich counts at the Spirit (Golombek, Crumpler, et al., 2006) and Phoenix landing sites (Golombek, Huertas, et al., 2012) and was inferred from the radar brightness (section 7) at the InSight landing site (Golombek et al., 2017; Putzig et al., 2017). The ~1% workspace rock abundance meets the requirement for deploying the instruments (<10%) and so posed no problem in placing the instruments in their optimal positions (maximum distance from the lander and each other).

Prior to landing, fragmentation theory (Charalambous, 2014) was also used to model the particle size‐frequency distribution of the regolith (including the rock abundance) based on the rocks and craters measured in HiRISE images (Golombek et al., 2017). Two fragmentation negative binomials were fit to all rocks measured in the landing ellipse as well as all rocks between 1.5 and 2.25 m diameter, because this is the size range that excludes many false positive detections used to define the CFA in 150 m by 150 m counting areas (Golombek et al., 2017; Golombek, Huertas, et al., 2012). These fits are shown in Figure 9 and are similar to the Phoenix and Spirit landing site rock size distributions for diameters smaller than about 1 m (Golombek et al., 2017). The rock distributions measured from the InSight lander generally fall in between those two landing sites and have a similar slope (Figure 10), which argues that the fragmentation theory predictions regarding rock abundance at the landing site are correct (Charalambous et al., 2019).

## 6 Slopes

Engineering constraints on slopes (<15°) at the landing site were evaluated at ~100 m length scale relevant to the radar tracking of the surface (actually at 84 m length scale) and at 1–5 m relevant to lander stability at touchdown and instrument deployment. Initially, slopes at 100 m length scale were evaluated in MOLA data, and later in digital elevation models (DEMS) made from stereo High‐Resolution Stereo Camera (HRSC) (Gwinner et al., 2016), CTX, and HiRISE images (Fergason et al., 2017). All the data show the InSight landing site to be among the smoothest landing sites on Mars at this length scale (Golombek et al., 2017).

**Figure 11:**
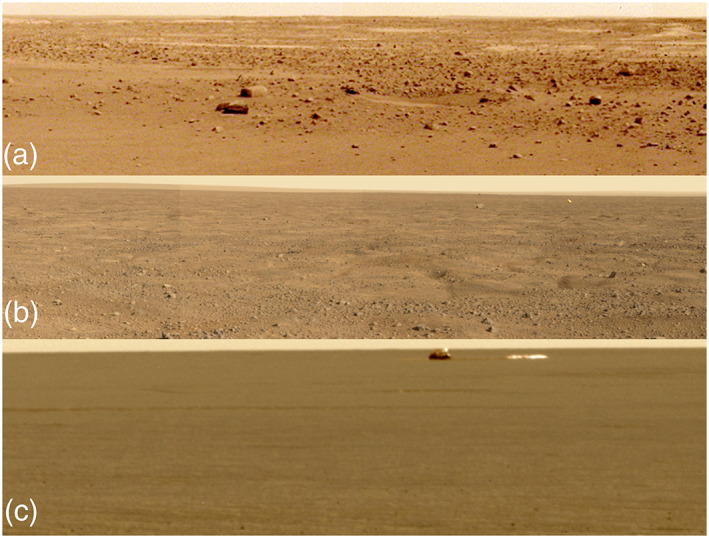
Portions of surface panoramas from the InSight (a), Phoenix (b) and Opportunity (c) landing sites, which are the three smoothest at 100 to 5 m length scales. Note similar roughness at these length scales as measured prior to landing in HiRISE DEMs and photoclinometry, and SHARAD. All mosaics have been contrast stretched and are not true color. The InSight panorama was acquired on Sol 14, the Phoenix image was acquired on Sol 15, and the Opportunity mosaic was acquired on Sol 21.

Relief and slopes at 100 m scale were estimated by extrapolating individual MOLA shot points along track from 1.2–0.3 km to 100 m from the Allen deviation and Hurst exponent, under the assumption of self‐affine statistics (Anderson et al., 2003). Root‐mean‐square (RMS) slopes and the Allen deviation (relief) at 100 m length scale for the landing location averaged in 12 km bins are 0.4° and 0.7 m, respectively, which is among the lowest measurable in the ellipse (Golombek et al., 2017). The MOLA pulse spread, which is sensitive to the RMS relief within the ∼75 m diameter laser shot point after removal of regional slopes (Garvin et al., 1999; Neumann et al., 2003) is 0.9 m at the landing site, averaged in 15 km bins; this is equivalent to a slope of <1° over 75 m. One hundred meter slopes within a 1 km square area of the lander in the HiRISE DEM indicate an RMS slope of 1.0°; CTX RMS slopes within a 1 km square area of the lander at this length scale are 1.4°. All of these measures indicate an exceptionally smooth surface that is consistent with the low relief surface and flat horizon, which is comparable to the smoothest landing sites at this length scale (Figures 1 and 11).

To estimate slopes at the 1–5 m length scale across the landing site, eight HiRISE DEMs (Fergason et al., 2017) were used to tune the photoclinometry (shape from shading) of 56 HiRISE images (Beyer, 2017). At the meter length scale, the smooth terrain in the landing site is extraordinarily smooth with the RMS slope in the DEMs of 3.9° and 0.4% of the area with slopes >15° (Fergason et al., 2017). Photoclinometry 2 m bidirectional slopes at InSight are less than those at the Viking, Pathfinder, Spirit, or Curiosity landing sites with <0.5% of the surface exceeding 15° (Beyer, 2017). At the 5 m length scale, RMS slopes of the InSight landing site are 2–3° and comparable to the smoothest landing sites (Phoenix and Opportunity) (Golombek et al., 2017) (Figure 11). Because InSight landed in one of the HiRISE DEMs (InSightE17_C in Fergason et al., 2017), the RMS slope of the area within a 1 km square area of the landing location at 2 and 5 m length scale was calculated to be 2.9° and 2.4°, respectively (3.6° for the entire DEM; Fergason et al., 2017) with no area >15°, which is smoother than average for the smooth terrain (Fergason et al., 2017) and for the ellipse (Golombek et al., 2017). A DEM of the workspace from stereo lander images at a resolution of 1 mm per elevation posting was used to calculate the 2 m RMS slope of smooth terrain on the floor of *Homestead hollow*. The 2 m RMS slope of the workspace is 1.6°, which is less than the HiRISE slope of the area within 1 km of the lander and consistent with the floor of *Homestead hollow* being much smoother than the surrounding rougher terrain (e.g., Figures 1 and 4). Slopes within the workspace were not a factor in the placement of the instruments on the surface.

## 7 Radar

As part of the landing site evaluation process, two radar data sets were evaluated to assess radar reflectivity to confirm the descent altimeter and velocimeter on InSight would function properly, that the surface was load bearing, and that the surface roughness was within the capabilities of the lander (Golombek et al., 2017; Putzig et al., 2017). Arecibo 12.6 cm radar imaging (Harmon et al., 2012) and same‐sense polarization were evaluated because the wavelength is most similar to the InSight C‐band (7 cm) altimeter. Arecibo radar images with a lateral resolution of ~3 km show a radar reflective surface with moderate backscatter strength and no anomalous returns that might compromise the altimeter. In addition, the backscatter strength is inconsistent with rock‐poor, porous material (Putzig et al., 2017), suggesting that the landing site is load bearing with average normal reflectivity and bulk density (>1,300 kg/m^3^) (e.g., Golombek et al., 1997). The Arecibo same‐sense polarization echoes of the InSight ellipse, which are most strongly affected by the small‐scale surface roughness and rock abundance, are higher than the Viking landing sites and similar to surfaces adjacent to Halemaumau crater on Kilauea volcano whose rocks were emplaced by phreatomagmatic eruptions (Campbell, 2001). Rock size‐frequency distributions for diameters <10 cm for these surfaces are steeper than exponential model rock distributions for Mars (and the Viking lander sites) (Campbell, 2001; Craddock & Golombek, 2016), suggesting that the InSight landing site has a greater abundance of 2–10 cm rocks than the Viking sites (Putzig et al., 2017) as discussed in section 5.

SHARAD data were used to determine a roughness parameter from the ratio of echo power integrated over a range of incidence angles to the peak echo power and is related to the RMS slope of the surface at scales of 10 to 100 m (Campbell et al., 2013; Putzig et al., 2017). The SHARAD‐derived roughness parameter for the InSight landing site (~3) overlaps that for the Opportunity and Phoenix landing sites, indicating the InSight landing site would be similar to these two sites in roughness at the 10 to 100 m length scale. This result is consistent with the similar HiRISE and MOLA RMS slope results at 5 and 100 m length scales discussed in section 6.

InSight landed safely and entry, descent, and landing reconstructions indicate the radar altimeter and velocimeter worked properly (Karlgaard et al., 2020; Maddock et al., 2020) and that the landing site is radar reflective as indicated by Arecebo radar images. The surface where InSight landed is clearly load bearing as the lander did not sink appreciably into the surface as indicated by both the radar reflectivity (and inferred bulk density) and the thermophysical properties (section 2). The SHARAD roughness at 10 to 100 m length scale is consistent with the similar HiRISE and MOLA RMS slope results at 5 and 100 m length scales discussed in section 6. The similarity in roughness at these length scales is also evident in the lack of relief in the panoramas of the landing sites (Figure 11). Finally, rock size‐frequency distributions measured near the InSight lander show a pebble rich surface with much higher abundance and a steeper distribution for rocks smaller than 8 cm than either Viking landing site or the exponential models and are similar to the pebble rich surfaces at the Spirit and Phoenix landing sites (section 4) as predicted by the Arecibo same‐sense polarization echoes.

## 8 Corinto Secondaries

During the evaluation of prospective InSight landing sites, it was recognized that secondary craters from the fresh rayed crater Corinto, about 700 km northeast were omnipresent in the region (Golombek et al., 2017). Corinto is among the youngest rayed craters on Mars, having formed after a young 2.5 ± 0.2 Ma Elysium lava flow but before Zunil secondaries (0.1–1 Ma) (Bloom et al., 2014; Golombek, Bloom, et al., 2014; Hundal et al., 2017). Corinto secondaries have distinctive bright ejecta, and their density was estimated by comparison with samples of measured density in 2.5 km by 2.5 km square areas in HiRISE images of the landing sites to help understand potential risks to the spacecraft. Five density classifications were defined, with the middle three covered by 0.6%, 1.6%, and 5.1% of secondary craters (Golombek et al., 2017). InSight landed within a 2.5 km square area with a medium‐high density (~5% of the surface covered by secondary craters), immediately adjacent to a sample with low density (~0.6%), so it is likely that Corinto secondaries would be common nearby. To evaluate the risk to landing on Corinto secondaries, an extensive measurement campaign of their morphometry was undertaken in HiRISE DEMs and photoclinometry. Measurements of over 900 secondaries showed very low depth/diameter ratios (0.05) that had parabolic and conical cross‐sectional shapes with slopes well below the 15° lander engineering constraint (Golombek et al., 2017).

**Figure 12:**
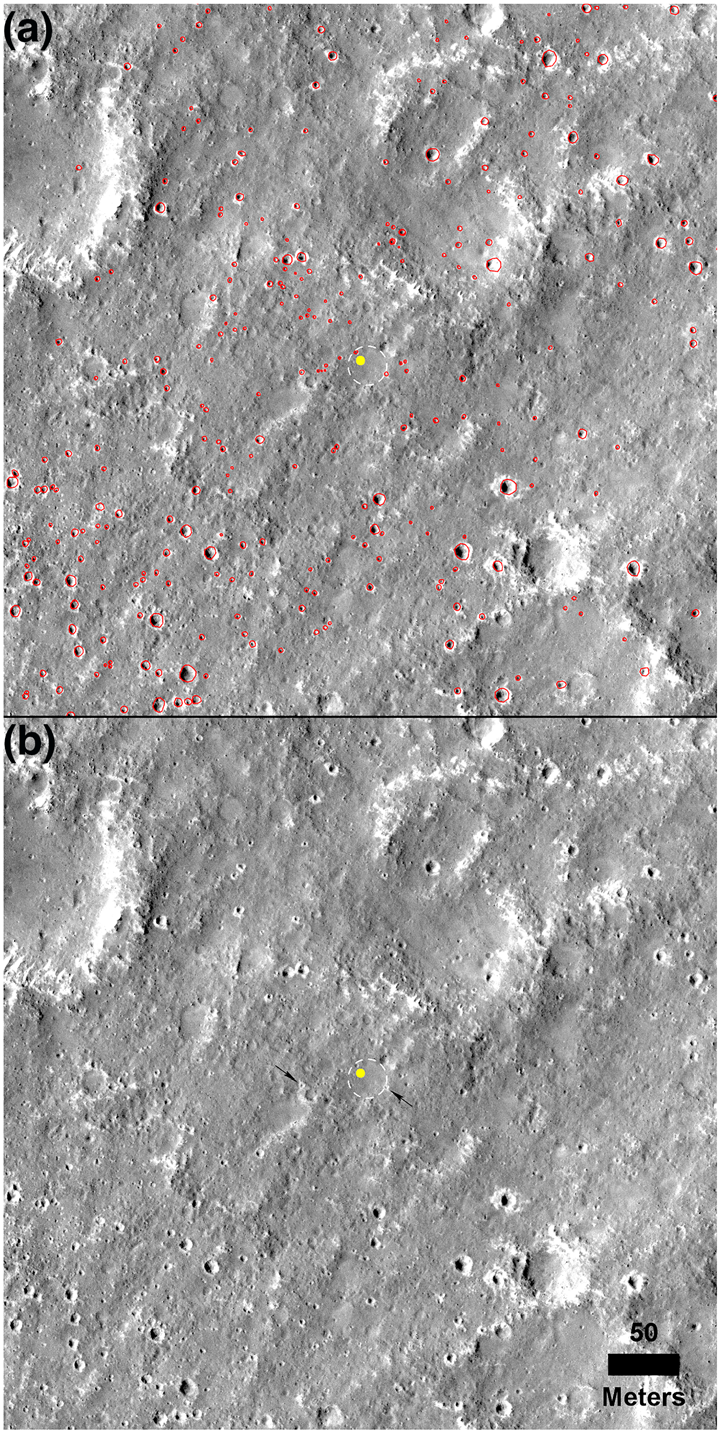
Square portion of HiRISE image (0.5 km sides) centered at the InSight lander (yellow dot). (a) Corinto secondaries circled in red. (b) unannotated image. Note distinctive bright ejecta of Corinto secondaries. All fresh craters with distinctive bright ejecta or craters with similar morphology (fresh bowl shape) but little or no bright ejecta (typical of very small craters) were measured down to 2 m diameter. HiRISE image ESP_036761_1845_RED taken before InSight landed. North is up. Note Corinto secondary crater, *Corintito*, 20 m to the southeast (Figure 1a) and *Corintitwo*, ~40 m to the west‐southwest of the lander are pointed out with black arrows in (b). Dashed line is the rim of *Homestead hollow*.

The diameter of all fresh craters within a 500 m square area centered on the lander was measured in a HiRISE image to determine the areal coverage (density) of Corinto secondaries (Figure 12). Results show that 2% of the surface in this area is covered by Corinto craters. This measured abundance of secondaries is consistent with estimates made in 2.5 km square areas prior to landing.

Because Corinto secondaries are expected to be common based on their density in HiRISE images, it is not surprising to have two Corinto secondaries within view of the lander. One Corinto secondary, dubbed *Corintito*, is located about 20 m to the southeast, just beyond the smooth terrain of *Homestead hollow* (Figures 1 and 12). Another Corinto secondary, dubbed *Corintitwo* is located about 40 m to the west‐southwest of the lander. In HiRISE images, both have the bright ejecta characteristic of Corinto secondaries (Figure 12). From InSight, only the rim of *Corintitwo* can be seen. However, *Corintito* is close enough to reveal a shallow bowl shape with a sandy interior. It appears fresh, with no bedforms, and its sandy interior suggests that no subsequent impacts have deposited rocks on it. The sandy interior suggests that the impact occurred in dominantly sandy material with few rocks. The diameters of *Corintito* and *Corintitwo* are both ~3 m measured in the HiRISE image and as estimated from lander images.

**Figure 13:**
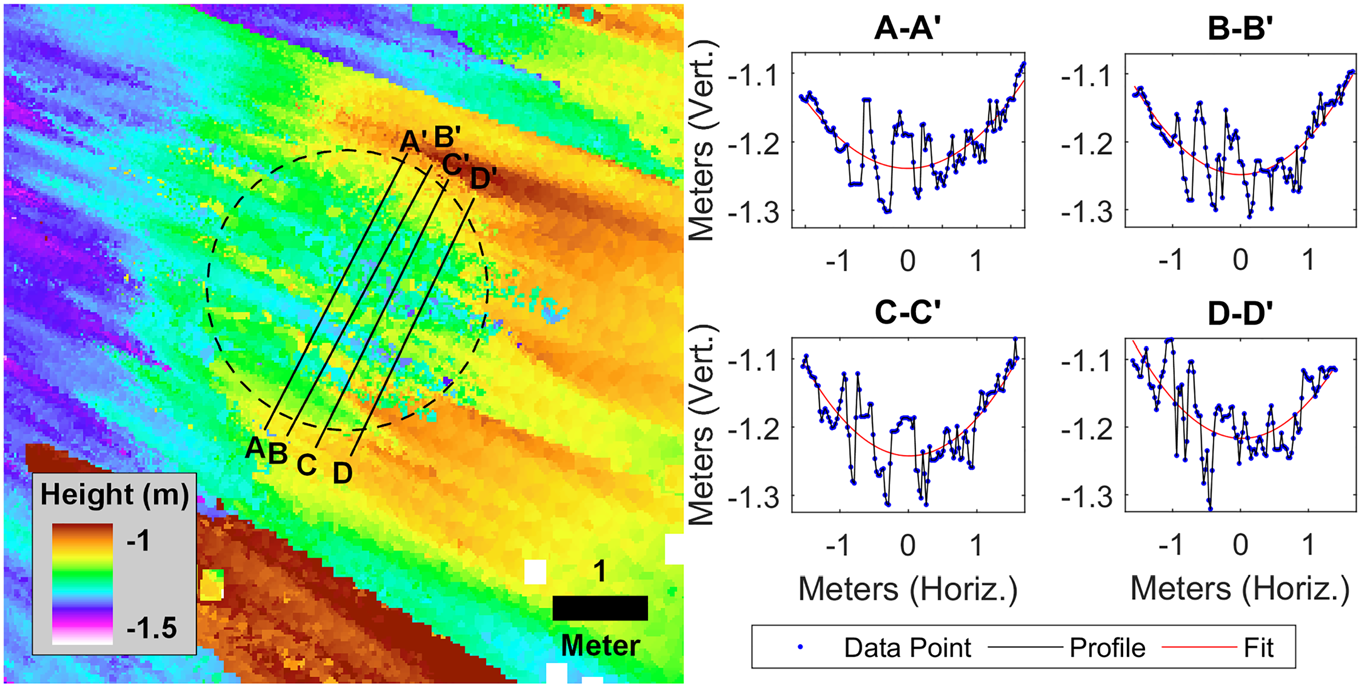
Digital elevation model and topographic cross sections of *Corintito* crater located about 20 m southeast of the InSight lander, which is shown from the lander in Figure 1 and in HiRISE in Figures 3 and 12. The depth/diameter ratio is 0.05, the crater shape is parabolic, and the inner wall slopes are generally 1–5°, with a maximum of around 10°, all of which are similar to morphometric measurements made from HiRISE images. Topographic elevation postings (2.5 cm) in the profiles are the blue points; the red lines are second degree polynomial least squares fits. Note the streakiness (noise) in the elevations is downrange from the camera and thus due to range error. However, elevations perpendicular are dominated by camera resolution and thus relative elevations along the profiles are reasonably accurate.

A preliminary DEM of *Corintito* from lander stereo images shows the crater has a maximum depth of 14.7 cm, which for a 2.95 m diameter indicates a depth/diameter ratio of 0.05 (Figure 13). In addition, the shape of the crater appears parabolic with steeper slopes near the rim. Parabolic models of a crater with a depth/diameter ratio of 0.05 yield maximum wall slopes of <11°, with most interior slopes of <5° (1–5°) (Golombek et al., 2017). Maximum near rim wall slopes for *Corintito* are around 10° with most interior slopes of 1–5°. In summary, observations from the surface of the areal density of Corinto secondaries, their depth/diameter ratio, shape, and interior slopes all agree with expectations prior to landing.

## 9 Regolith Thickness

**Figure 14:**
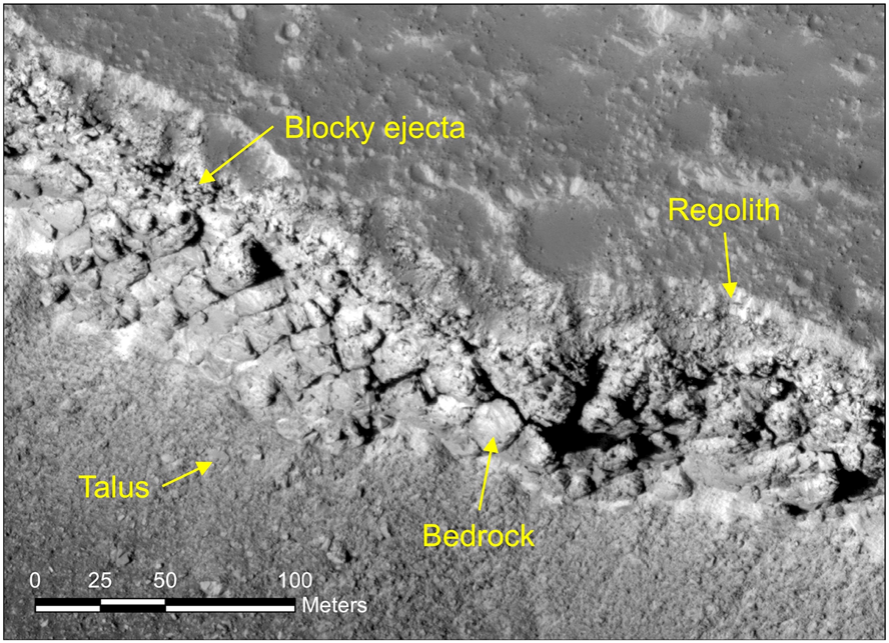
HiRISE image of steep exposed scarp of Hephaestus Fossae in southern Utopia Planitia at 21.9°N, 122.0°E. The image shows cratered plains in the upper right, with the scarp exposing ∼10 m thick, relatively fine grained regolith that grades into blocky, coarse breccia that overlies strong, jointed bedrock below. The talus is below the bedrock in the exposed scarp. This near‐surface stratigraphy is indicated by rocky ejecta craters, concentric or nested craters, and fragmentation theory for the InSight landing site (Golombek et al., 2017; Warner et al., 2017). Portion of HiRISE image PSP_002359_2020.

A requirement for the landing site is 3–5 m of poorly consolidated, relatively fine grained material conducive for penetration by the HP^3^ mole (Golombek et al., 2017). Measurements of the onset diameter of RECs and nested or concentric craters indicate that a near‐surface fragmented regolith 3–17 m thick exists at the landing site (Warner et al., 2017), which is also indicated by fragmentation theory (Golombek et al., 2017). HiRISE images of nearby Hephaestus Fossae show a relatively fine grained near‐surface regolith that grades into coarse breccia overlying intact bedrock (Figure 14) and are consistent with expectations from fragmentation theory (Golombek et al., 2017). Thickness variations of the regolith were estimated from the fraction of RECs versus all fresh craters and the fraction of non‐RECs versus all fresh craters 30–60 m diameter (Golombek, Grott, et al., 2018). Results show a higher percentage of rocky versus non–rocky craters of this size are present in the west central portion of the ellipse, suggesting a generally thinner regolith there than elsewhere. InSight landed in a 5 km by 5 km square grid where 60% of all craters are RECs and 40% of all craters are non‐RECs <60 m diameter (Golombek, Grott, et al., 2018), suggesting a somewhat thinner than average regolith beneath the lander.

**Figure 15:**
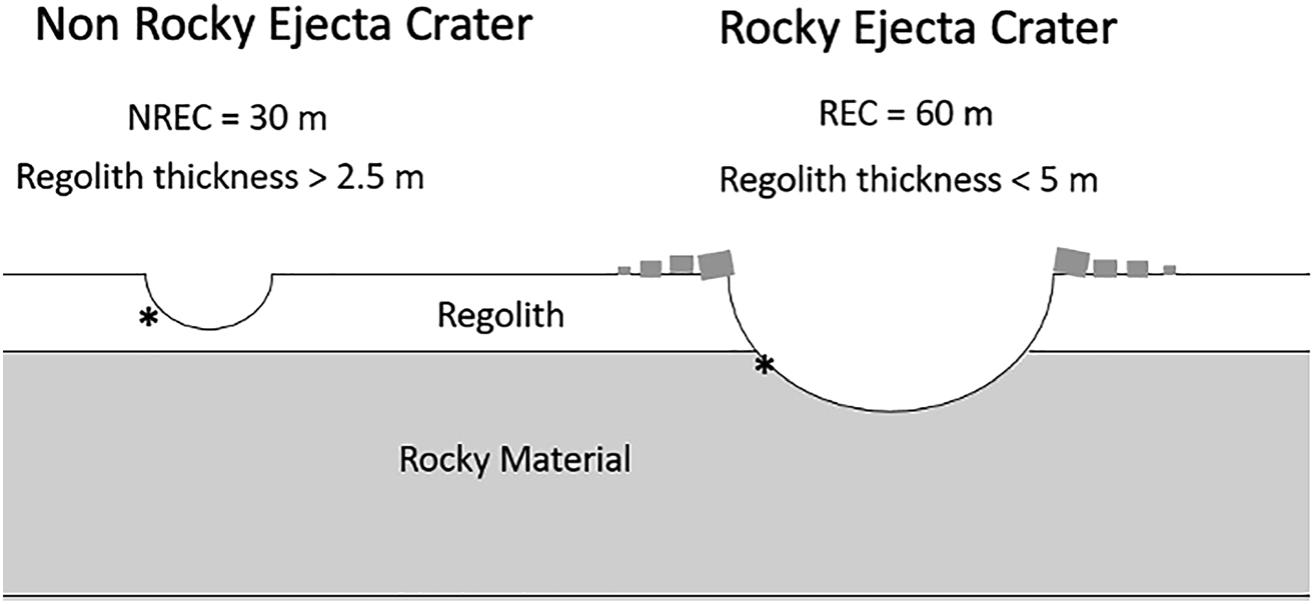
Conceptual cross section of a 30 m diameter non–rocky ejecta crater (left) and a 60 m diameter rocky ejecta crater (right). Ejecta is sourced from 0.084 times the crater diameter (marked by the asterisk, *), so the non‐REC indicates the fragmented regolith is at least 2.5 m thick. The REC indicates the regolith must be thinner than its ejecta source depth of 5 m. The actual thickness of the regolith must be between these two (i.e., >2.5 and <5 m).

To map the thickness of the fragmented regolith, the location, diameter, and the presence or lack of rocks of all fresh craters in the landing ellipse were mapped (Golombek, Grott, et al., 2018; Warner et al., 2017). The continuous ejecta from a simple crater is sourced from a depth of about 10% of the transient crater diameter or about 0.084 times the diameter of the final crater (Melosh, 1989). A fresh non‐REC indicates that the fine‐grained regolith must be at least as thick as 0.084 times its diameter. This is a measure of the minimum thickness of the regolith at that point (Figure 15). Conversely, a fresh REC indicates that the intact basalt (or possibly very coarse basalt breccia) is at 0.084 times its diameter beneath the crater and that the fine‐grained regolith must be less than this depth (Warner et al., 2017). As a result, the actual depth of the base of the fine‐grained regolith must be below the ejecta source depth from non‐RECs and it must be above the ejecta source depth from RECs (Figure 15). Contouring the non‐REC source depth and the REC source depth separately shows a similar pattern of thick and thin regolith thickness across the ellipse as estimated from the percentage of RECs and non‐RECs but also produces overlaps of inconsistent depths in areas with closely spaced RECs and non‐RECs.

To avoid this, a Delaunay triangulation algorithm finds a triangular plane of like closest points such that no points are inside the circumcircle of any triangle (Bern & Eppstein, 1992). The algorithm finds the three vertices of the closest non‐REC and REC source depths and tests to be sure that no REC source depth triangular plane is above a nearby non‐REC source depth triangular plane. The Delaunay triangulation maximizes the angles of the triangles to avoid thin slivers and determines the three‐dimensional planar surfaces that are confidently in the fine‐grained regolith (minimum thickness) and the bedrock.

**Figure 16:**
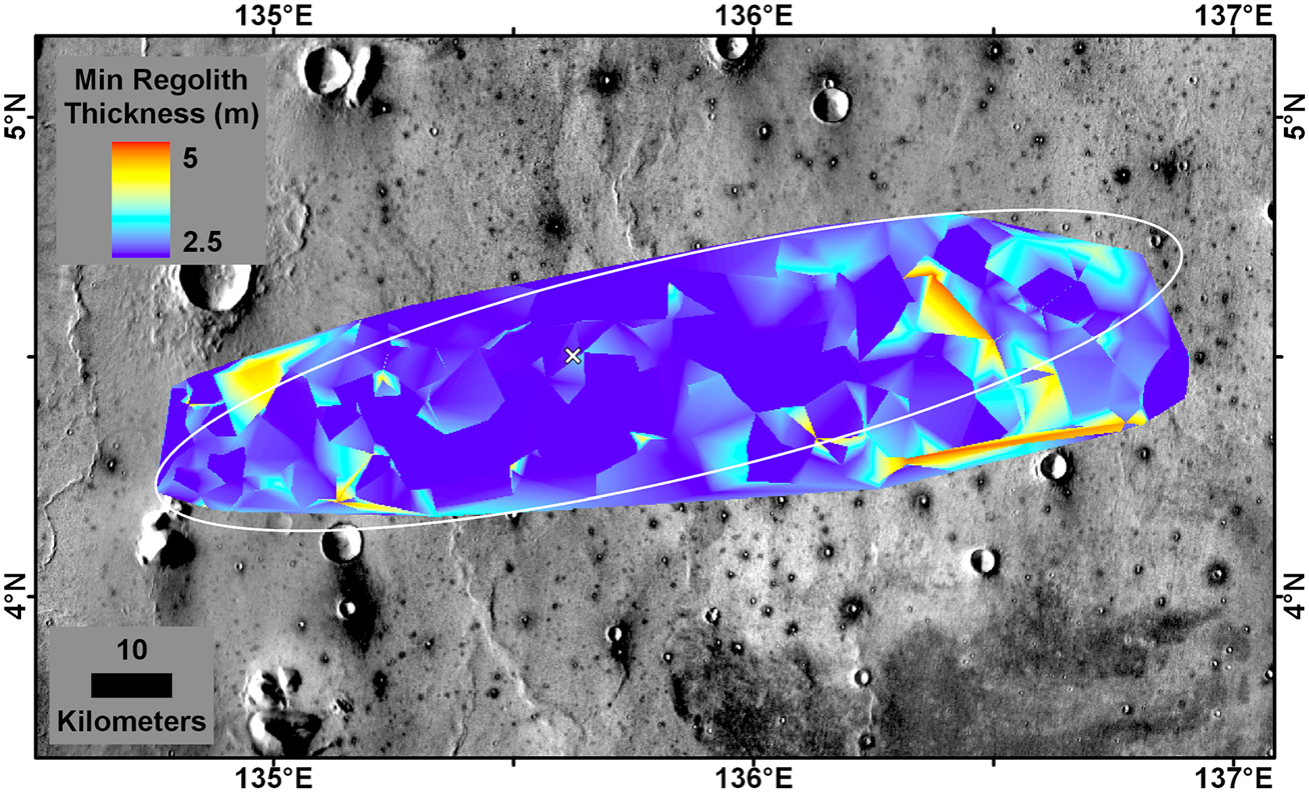
Delaunay triangulation of the source depth for non‐RECs 30–60 m diameter (Warner et al., 2017), which is the minimum regolith thickness at the InSight landing ellipse (in white). The minimum regolith thickness beneath the InSight lander (white X) is 2.6 m. We selected craters 30–60 m diameter to focus on 3–5 m depths relevant to the HP^3^ mole and because almost all larger craters (e.g., >200 m) are RECs. Latitude and longitude in planetocentric degrees; minimum regolith thickness. Background is the THEMIS daytime mosaic in which dark halo craters have higher thermal inertia from rocky ejecta. Note generally north trending wrinkle ridges.

**Figure 17:**
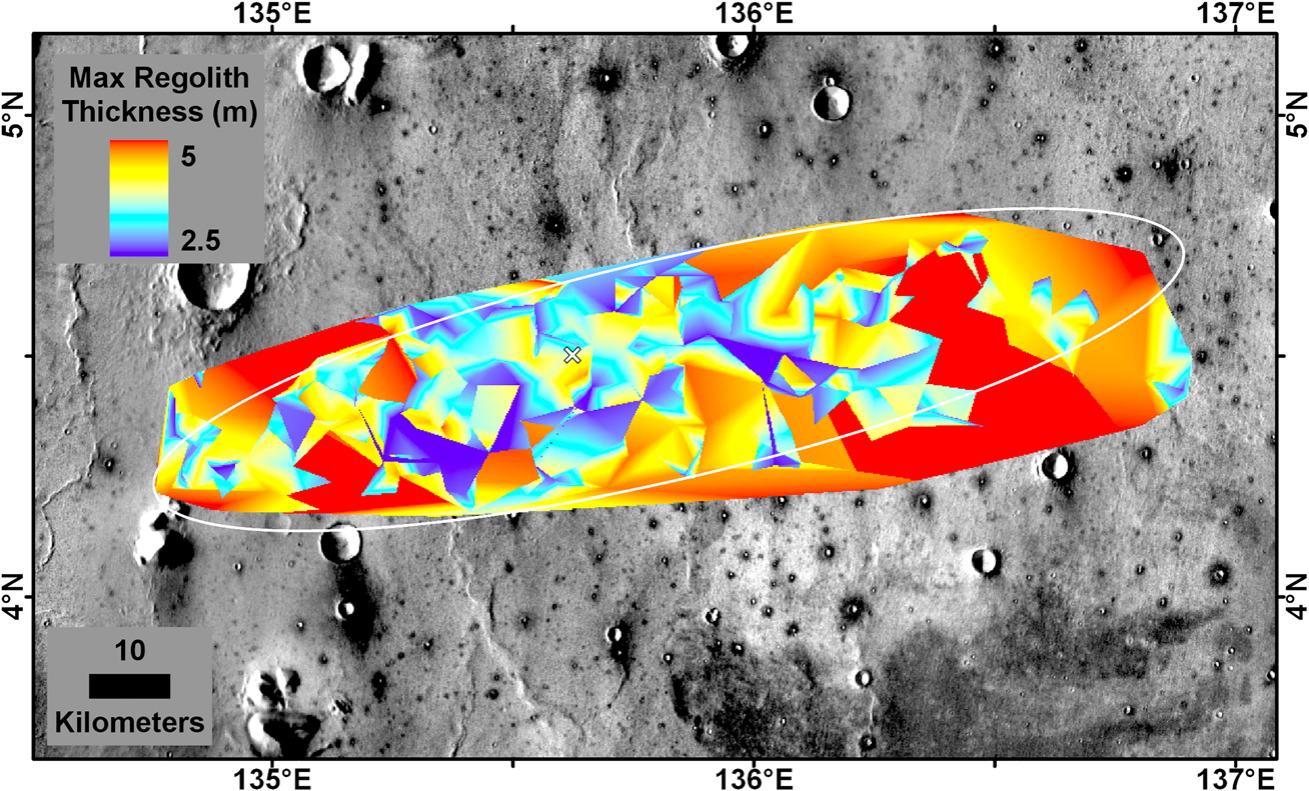
Delaunay triangulation of the source depth for RECs 30–60 diameter (Warner et al., 2017), which is the depth to rock in the InSight landing ellipse (in white). The base of the regolith must be above this depth so this is the maximum regolith thickness. The depth to rock beneath the InSight lander (white X) is 3.9 m. Latitude and longitude in planetocentric degrees; depth to rock. Background is the THEMIS daytime mosaic in which dark halo craters have higher thermal inertia from rocky ejecta.

**Figure 18:**
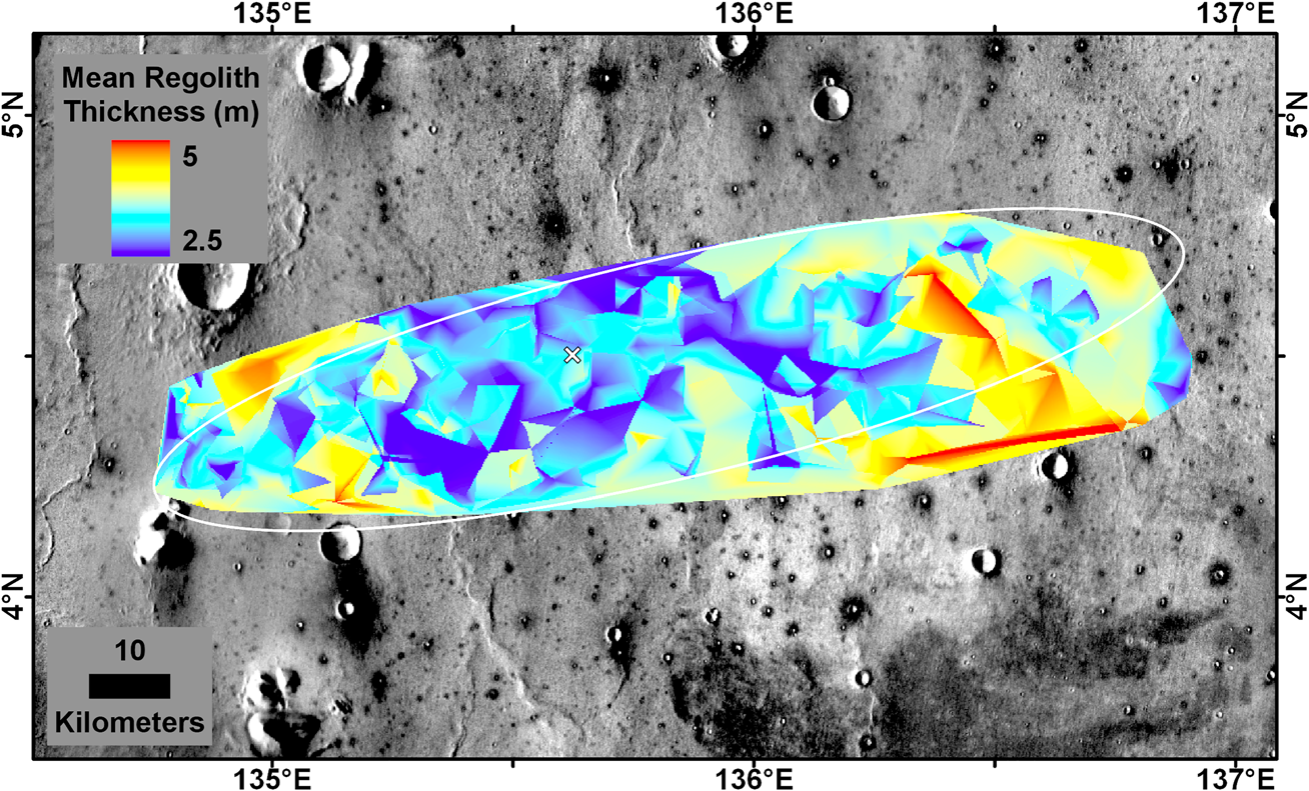
Delaunay triangulation of estimated regolith depth assuming that the base of the regolith is half way (mean) between the minimum regolith thickness (Figure 16) and the depth to the rock (Figure 17) based on the ejecta source depth of 30–60 m diameter fresh non‐RECs and RECs (Warner et al., 2017). The thickness of the regolith beneath the InSight lander (white X) is 3.3 m based on this determination. Latitude and longitude in planetocentric degrees; ellipse in white. Background is the THEMIS daytime mosaic in which dark halo craters have higher thermal inertia from rocky ejecta.

The Delaunay triangulation source depth of all fresh non‐RECs <60 m diameter is shown in Figure 16. This represents the minimum fine‐grained regolith thickness and shows that 86% of the ellipse and most of the central part has a minimum regolith thickness of 3 m. The area beneath the lander has a minimum fine‐grained regolith thickness of 2.6 m. The Delaunay triangulation source depth of all RECs <60 m diameter also shows the rock layer is shallower in the center of the ellipse (Figure 17). The area beneath the lander has rock at 3.9 m depth. As a result, the thickness of the fine‐grained regolith must be between 2.6 and 3.9 m beneath the lander. If the actual base of the fine‐grained regolith is halfway between the minimum regolith depth and the rock depth, then ~70% of the ellipse has a regolith thickness of >3 m (Figure 18); the thickness of the regolith beneath the lander would be 3.3 m.

Observations near the lander are consistent with an ~3 m thick fine‐grained regolith (Warner et al., 2019, 2020). The nearest relatively fresh crater is about 315 m to the southwest of the lander and is 45 m diameter. It is not a REC, indicating the regolith is at least 3.8 m thick at this location, which is consistent with the ellipse wide estimate. The closest REC is 100 m diameter about 400 m to the east‐southeast (Golombek, Warner, et al., 2020), indicating that rock is at 8.4 m depth. The 3 m diameter *Corintito* crater has a sandy interior, indicating that it impacted a sandy substrate at least 15 cm deep at 0.1–2.5 Ma. Finally, the preerosion diameter of *Homestead hollow* is estimated to be around 22 m (Warner et al., 2020). When it formed, its fresh crater depth would have been ~3.3 m, assuming a fresh depth/diameter ratio of 0.15 (Sweeney et al., 2018). Most of this would have been filled with sand since around 400–700 Myr (its crater retention age), which is also consistent with an ~3 m thick regolith beneath the lander (Grant et al., 2020). Finally, measured seismic velocities near the surface are consistent with unconsolidated relatively fine grained materials that are a few meters thick (Lognonné et al., 2020).

Even though there is strong evidence that an impact fragmented regolith at least 3 m thick exists beneath the lander, the HP^3^ mole has struggled to penetrate deeper than its length of 40 cm 1.5 years after landing (Hudson et al., 2020). Prior to landing, synthetic regoliths from fragmentation theory and mean free path statistics for observed surface rock abundances indicated a high probability of successfully penetrating at least 3 m for cases tested where the mole can move rocks <10 cm and divert around larger rocks if it encounters a rock at angles <45° (Golombek et al., 2017). After deployment of the HP^3^, the mole only partially penetrated before halting. Removal of the HP^3^ Support Structure revealed an open, steep sided pit without adjacent surface piles of soil (Golombek, Warner, et al., 2020). This suggests that the underlying soil is either low density and/or particularly porous that was compressed and allowed displaced surface material to drain down. Further, an illuminated wall of the pit shows horizontal resistant layers composed of fines and pebbles with vertical edges and overhangs suggesting cemented material with cohesions up to ~10 kPa (Golombek, Warner, et al., 2020; Hudson et al., 2020), similar to other strongly cemented soils on Mars typically referred to as duricrust (Christensen & Moore, 1992; Golombek, Haldemann, et al., 2008). To penetrate, the mole uses an internal hammer whose force must be resisted by friction provided by soil around the hull (Spohn et al., 2018). The leading hypothesis is that the regolith (including the duricrust) has not provided the necessary friction (Hudson et al., 2020). To aid the mole, the scoop on the end of the arm has both pinned the side, providing friction, and pushed the top of the mole so that it has hammered fully below the surface. This indicates that a rock or other obstruction did not impede the mole (Hudson et al., 2020). It is not known why the duricrust does not show up as a steep inertia contrast in the top few tens of centimeters (from the lack of orbital seasonal variation), although a combination of low density, thin low inertia surface materials, extremely small but highly cemented regolith particles, or unmodeled seasonal clouds are possibilities (Mueller et al., 2020; Piqueux et al., 2020; Vasavada et al., 2017).

## 10 Surface Alteration

Surface alteration by the InSight retropropulsive thrusters was expected as the plumes interact with the surface during landing (Golombek et al., 2017). Extensive modification beneath the lander was a concern for InSight during development, because the thrusters are pulsed, which leads to shock waves that fluidize soils leading to substantially more erosion than conventional jets (Mehta et al., 2011, 2013; Plemmons et al., 2008). The same pulsed thrusters on Phoenix excavated 5–18 cm of soil beneath the lander to uncover hard subsurface ice that prevented further erosion (Mehta et al., 2011). Furthermore, because the subsurface of the InSight landing site was selected to have 3–5 m of unconsolidated or poorly consolidated soils, it would be an issue if craters were eroded beneath the thrusters during landing that were large enough to alter surface topography under the footpads and thus destabilize the lander. An approximate model, based on the total amount of momentum imparted to the surface by the thrusters, was evaluated during development (Golombek et al., 2017). Results showed that craters were expected beneath the thrusters, but they would not be large enough to appreciably alter the surface topography beneath the lander footpads. Also, like Phoenix (Arvidson et al., 2009), it was expected that surface dust, sand, granules, and pebbles would be moved in the area around the lander but that this would not modify the surface sufficiently to impact instrument deployment (Golombek et al., 2017). Finally, because the surface of the InSight landing site is moderately dusty, it was expected that the thrusters would remove the dust and create a dark spot (Golombek et al., 2017) (also see section 4).

**Figure 19:**
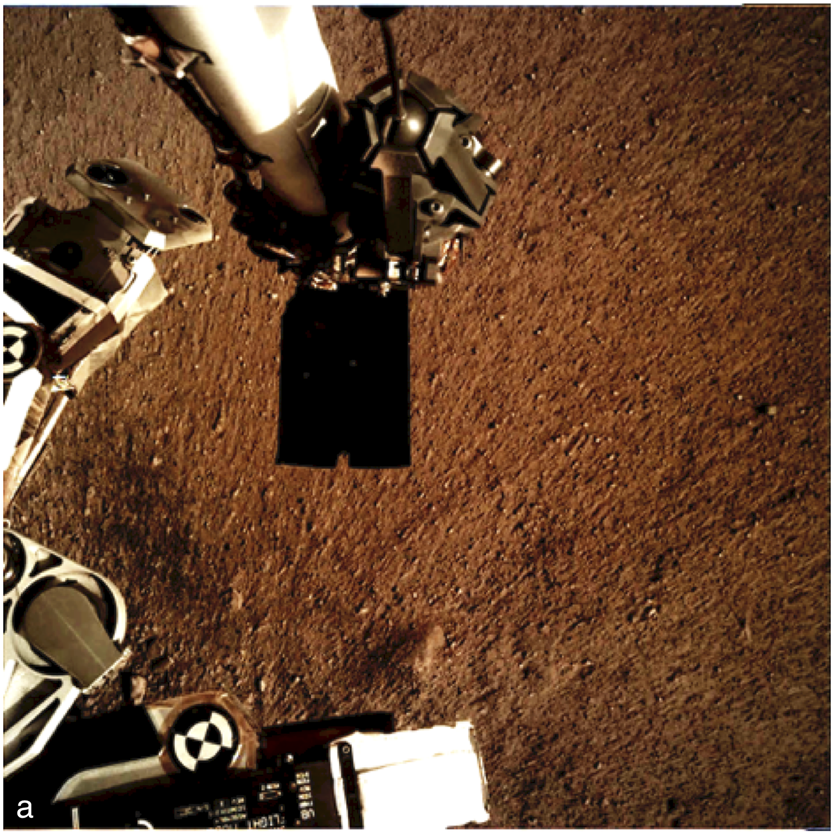
IDC image of the soil surface near the lander acquired on Sol 8. The radial striations in the soil and elongated hills that have pebbles at the lander‐facing ends suggest they protected the tails of material behind. The radial pattern suggests dispersal of unconsolidated sand by the retrorockets away from the lander. The relief of the ridges and grooves suggests that only millimeters of sand has been removed around the lander. The scoop at the end of the arm is the dark rectangle 7.1 cm wide in the center of the image.

**Figure 20:**
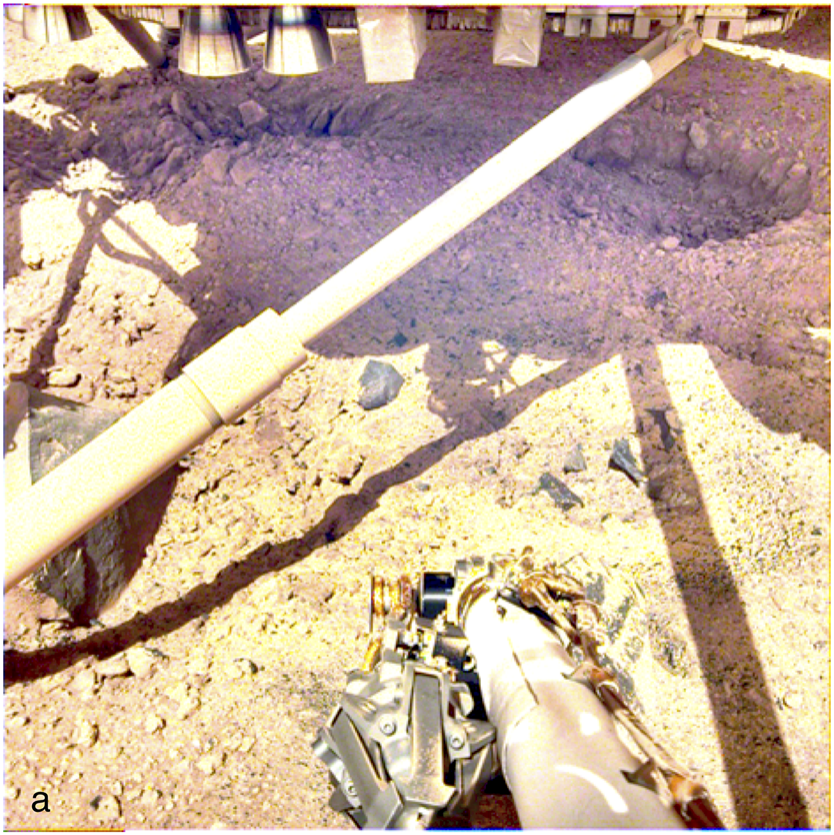
InSight IDC image under the lander showing pits ~50 cm diameter and ~10 cm deep dug by the pulsed retro rockets. The image was acquired on Sol 18 and has been contrast stretched to better show the surface in shadow beneath the lander.

The surface alteration observed around the InSight lander is consistent with expectations prior to landing (Golombek et al., 2017). As described more fully in section 4, HiRISE images taken about a week after landing show a large dark spot (Williams et al., 2019) (Figure 3) that extends to the north around 20 m (Figure 4). The spot is 35% lower in albedo than the surrounding surface but is more gradational and extended to the south (Golombek, Warner, et al., 2020). This darkening is similar in size and relative albedo change to the lander‐induced albedo changes around Phoenix and Mars Science Lander, all of which have been attributed to the removal of dust (Daubar et al., 2015; also see section 4). Around the lander, the surface has radial, millimeter‐scale ridges indicative of surface scour (Garvin et al., 2019) (Figure 19). Tails behind pebbles that extend away from the lander suggest scour by unconsolidated sand at the surface. Surface divots and elongated depressions leading to a 5 cm pebble indicate that it hopped and skipped ~1 m across the surface (Golombek, Warner, et al., 2020). These observations are consistent with the pulsed descent rocket exhaust removing bright surficial fine‐grained dust to create the dark spot and scouring dark loose sand and granules as expected from prelanding analysis (Golombek et al., 2017). Retrorockets excavated three pits ~50 cm diameter and ~10 cm deep beneath InSight (Figure 20). Material from the pits appears scattered across the surface, and one footpad is buried by this material. None of the pits extend to the footpads and are thus consistent with models of surface alteration done prior to landing. Furthermore, surface alteration by the thrusters did not negatively impact instrument deployment (Banerdt et al., 2020). These modifications are similar to those at the Phoenix landing site, where pulsed retrorockets removed dust, scoured the surface, and dug pits beneath the lander (Arvidson et al., 2009; Mehta et al., 2011).

## 11 Planetary Protection

The InSight landing site had to pass planetary protection requirements consistent with its designation as a Category IVa mission that could not go to a special region and carries no life detection instruments. For InSight, the landing site cannot have water or ice within 5 m of the surface (i.e., it cannot be a special region, e.g., Rummel et al., 2014), subsurface discontinuities, high concentrations of water bearing soils or minerals, or liquid water created by the HP^3^ mole that could mobilize a 50 nm particle (Golombek et al., 2017). Nearly complete high‐resolution image coverage of the landing ellipse revealed no geomorphic features that could be interpreted as due to liquid water or ice within 5 m of the surface (Golombek et al., 2017). In addition, there is no evidence that surface materials have high concentrations of water bearing minerals (e.g., Pan & Quantin, 2018; Pan et al., 2020). In fact, the formation of a fragmented regolith overlying basalt flows was viewed as a barrier for the generation of special regions and no subsurface reflectors were identified in SHARAD data (e.g., Putzig et al., 2017). Finally, orbital data show that the surface was only affected by impact, mass wasting, and eolian processes (Golombek et al., 2017), and a review of the literature revealed no other proposed aqueous activity in the region since the basalt was emplaced.

The evaluation of the surface prior to landing with regard to planetary protection requirements is consistent with observations from the surface after landing. Observations of the geology of the surface from the lander only show evidence for impact, mass wasting, and eolian processes (Golombek, Warner, et al., 2020). No geomorphological evidence for subsurface water or ice has been found. Rocks are dark and fine grained, consistent with mafic, aphanitic basalts. Finally, surface investigations are consistent with surface materials composed of poorly sorted, dominantly sandy soils with lesser granule, pebbles and rocks that formed via impact, mass wasting and eolian processes (section 9) (Golombek, Warner, et al., 2020).

## 12 Comparison to the Gusev Cratered Plains

The interpretation of the smooth plains in the InSight landing ellipse as being composed of an impact generated regolith overlying basalt flows being similar to the Gusev cratered plains from the Spirit rover traverse suggested that the landing sites would be dominated by impact craters in various stages of degradation and that hollows (filled craters) would be common (Golombek et al., 2017; Golombek, Grott, et al., 2018; Sweeney et al., 2018; Warner et al., 2017). In this section, we assess the similarities between the two sites.

Prior to landing, the smooth plains of the InSight landing site were interpreted to be a 3–10 m thick, impact generated regolith underlain by Hesperian to Early Amazonian basaltic lava flows based on the following observations. Geologic mapping in CTX images showed volcanic vents and flow fronts that partially fill large craters (Golombek, Grott, et al., 2018). HiRISE images of Hephaestus Fossae in southern Utopia Planitia (Figure 14) show ~10 m of regolith with few rocks over coarse breccia and steep, jointed bedrock below (Golombek et al., 2017; Warner et al., 2017). Rocks in the ejecta of fresh craters ~0.5–2 km diameter indicate competent rock ~5–200 m deep with weaker material above and below (e.g., Golombek et al., 2017; Warner et al., 2017). Shallow exposed outcrops in fresh crater walls have mafic mineral spectra consistent with basaltic material (Pan & Quantin, 2018; Pan et al., 2020). Wrinkle ridges on the plains (Figure 16) have been interpreted as fault‐propagation folds where slip on a thrust fault at depth results in asymmetric folding in weakly bonded but strong layered material (such as basalt flows ∼200–300 m thick) near the surface (e.g., Golombek & Phillips, 2010; Mueller & Golombek, 2004).

Observations of 100 m scale impact craters in the InSight landing area showed that they are in a variety of degradation states from fresh, bowl shaped craters to nearly, flat filled in circular hollows (Warner et al., 2017; Sweeney et al., 2018). Analysis of the size‐frequency distribution of these craters and the morphometry of the different degradational states from DEMs yielded timescales and amounts of erosion. Results show that the crater degradation rates are slow (10^−2^ to 10^−4^ m/Myr) (Sweeney et al., 2018; Warner et al., 2020) and consistent with a surface shaped predominantly by impact, mass wasting, and eolian processes, similar to other Hesperian surfaces on Mars (e.g., Golombek, Grant, et al., 2006; Golombek, Warner, et al., 2014). Constraints on the age of the volcanic flows from the size‐frequency distribution of craters indicates Hesperian ages for craters larger than 5 km (Golombek, Grott, et al., 2018), but craters smaller than 2 km indicate volcanic resurfacing in the Early Amazonian (~1.7 Ga) (Warner et al., 2017, 2020; Wilson et al., 2019). In addition, craters smaller than 150 m diameter fall along a −2 power law slope (Warner et al., 2020; Wilson et al., 2019) indicating that they are in equilibrium with surface processes eroding and filling them in (i.e., new craters form at about the same rate at which they are destroyed) (Hartmann, 1984). Finally, our analysis of regolith thickness (section 9) shows that most of the ellipse has a regolith 3–5 m thick. These orbital observations are consistent with our observations and interpretations made from the lander (Golombek, Warner, et al., 2020). In particular, within view of the lander are a large number of craters, with many appearing as hollows (nearly flat, filled in craters).

**Figure 21:**
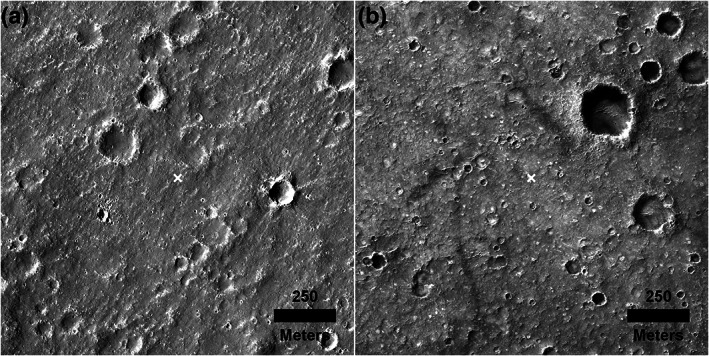
HiRISE images of the InSight (a) and Spirit (b) landing sites at the same scale. The white crosses (X) show the location of the landers. Note smoother surface and more degraded craters at the InSight landing site compared with the Spirit landing site. Bonneville crater is the largest, freshest crater (210 m diameter) with continuous rocky ejecta blanket investigated by the Spirit rover to the northeast. Compare with the freshest crater near InSight, which is the 100 m diameter REC around 400 m to east. HiRISE image (a) is ESP_036761_1845; HiRISE image (b) is PSP_001777_1650.

Observations of the Gusev cratered plains during the Spirit rover traverse (Grant et al., 2004; Grant, Arvidson, et al., 2006; Golombek, Crumpler, et al., 2006) also indicate a similar origin as an impact generated regolith about 10 m thick that overlies Hesperian‐Early Amazonian basalt flows that has been degraded by mass wasting and eolian processes. The Gusev cratered plains are named because of the ubiquity of craters in a variety of degradation states and the preponderance of hollows, which are low relief circular features with relatively smooth soil filled interiors surrounded by rockier terrain (Figure 1). The rocks are olivine basalts (McSween et al., 2006), and field observations identified vesicular clasts and rare scoria similar to original lava flow tops, consistent with an upper inflated surface of lava flows with adjacent collapse depressions (Golombek, Crumpler, et al., 2006). The measured size‐frequency distribution of impact craters >20 m diameter in Mars Orbital Camera images shows they also are on a −2 power law distribution (Golombek, Crumpler, et al., 2006), suggesting that they are in equilibrium with surface processes eroding them (Hartmann, 1984). Hollows down to ~2 m diameter measured from the Spirit rover while traversing from the landing site to Bonneville crater are continuous with the −2 power law distribution for larger craters but have a lower power law slope of −1.5 indicating that smaller craters are being destroyed more rapidly than larger ones. Two‐toned or patinated rocks and elevated ventifacts indicate eolian deflation of the Gusev cratered plains surface of ~5–25 cm and the transported sand filled the craters to form the hollows (Golombek, Crumpler, et al., 2006; Golombek, Grant, et al., 2006). Further constraints on the erosion of fines based on the rock distributions limits the total deflation from tens of centimeters to less than 1 m (Golombek, Grant, et al., 2006), which constrains the rate of erosion to <10^−3^ to 10^−4^ m/Myr (Golombek, Crumpler, et al., 2006; Golombek, Grant, et al., 2006). The size‐frequency distribution of craters >1 km in HRSC images for the Gusev surface indicate a Hesperian age of ~3.65 Ga (Greeley et al., 2005). Smaller craters (0.2–1 km) in higher‐resolution images follow a production function for a 2.2 Ga, Early Amazonian age (Wilson et al., 2020), consistent with younger resurfacing. The thickness of the regolith on the Gusev plains is estimated at around 10 m from the morphology and morphometry of the largest, freshest crater visited by the Spirit rover. Bonneville crater is a 210 m diameter, 10 m deep, flat floored fresh crater, with a rocky continuous ejecta blanket (Figure 21). Modification is limited to thin (few meters) drift deposits, and jumbled rocks make up the constant inward sloping walls, with the largest blocks (2.5 m) suggesting Bonneville crater formed in loose rubble ~10 m thick but may have excavated some rocks from deeper more intact rock (Grant et al., 2004; Grant, Wilson, et al., 2006; Golombek, Crumpler, et al., 2006).

The above descriptions of the InSight smooth plains and Gusev cratered plains clearly indicate that both sites share a similar origin as basalt flows with an impact generated regolith modified by mass wasting and eolian activity. Both sites contain impact craters in a variety of degradational states and have abundant hollows attesting to eolian infill. In this regard, the prediction that the InSight landing site would be similar to the Spirit landing site appears largely correct.

However, we also note interesting differences between the two sites that warrant further investigation. Side‐by‐side HiRISE images of both surfaces at the same scale show that the InSight surface is smoother and craters are more subdued and degraded and has far fewer fresh craters tens of meters in diameter than the Gusev cratered plains (Weitz et al., 2020) (Figure 21). The Gusev surface also has more eolian bedforms and more dark bedforms, suggesting that they are more recently active. Both InSight and Gusev crater ages indicate Hesperian surfaces that were resurfaced in the Early Amazonian. However, the erosion rates at the InSight landing site are 1 to 2 orders of magnitude faster than at the Gusev cratered plains, which could explain the smoother surface at InSight. Further research could help to better understand these differences. The existence of two landing sites on Mars that formed in a similar way offers a rare opportunity to better understand the cause of their differences.

The similar remote sensing properties and geologic settings of the InSight and Spirit landing sites do suggest that Hesperian lava flow surfaces on Mars develop fragmented regolith‐dominated surfaces modified by mass wasting and eolian processes with generally low thermal inertia (e.g., Golombek, Crumpler, et al., 2006; Golombek, Warner, et al., 2020; Grant, Arvidson, et al., 2006). In these terrains, the mechanically strong blocks of basalt bedrock are fragmented by impact but otherwise resist further significant breakdown by eolian activity (e.g., Rogers et al., 2018). On these types of surfaces, the sand produced by the impacts (Golombek, Charalambous, et al., 2018, 2020) is trapped in the craters, producing a surface dominated by sand with variable rock abundance and generally low thermal inertia (e.g., Golombek, Crumpler, et al., 2006; Golombek, Warner, et al., 2020; Rogers et al., 2018). This is in contrast to surfaces with higher thermal inertia that are composed of generally weak, friable clastic rocks that are easily eroded and cleared of comminuted material by eolian activity (e.g., Rogers et al., 2018). These surfaces generally have higher thermal inertia than those composed of mostly sandy regolith. Examples of weak, friable clastic rocks include the sulfate sandstones of Meridiani Planum that have been easily planed off parallel to the surface by eolian activity (e.g., Golombek, Warner, et al., 2014) and the rocks of the Columbia Hills that lack a regolith cover (Golombek, Grant, et al., 2006; Rogers et al., 2018). Future landings on Mars and comparisons to geologic interpretations of remote sensing data from orbit will further test and refine these relationships.

The successful prediction of both the surface characteristics important for landing safely (e.g., rocks, slopes, load bearing, and radar reflective) and the geologic setting (in this case basalt flows overlain by fragmented regolith) is only the third case (the other two being the Mars Pathfinder and Phoenix landing sites) where both have been correctly predicted from orbital remote sensing data. Part of this success could be due to having a similar site already explored on the ground (Spirit) and perhaps also to the fairly simple geologic setting (basalt with overlying regolith for Spirit and InSight). With regard to just the surface characteristics important for landing safely, the analysis of remote sensing data has correctly predicted the last six Mars landing sites (Mars Pathfinder, Mars Exploration Rover Spirit, Opportunity, Phoenix, MSL, and InSight; Arvidson et al., 2008; Golombek et al., 1997, 1999, 2005, 2017; Golombek, Grant, et al., 2003, 2012; Golombek, Haldemann, et al., 2008). A significant factor in the last three successful site selections has been the ability to detect rocks and slopes in HiRISE images and DEMs at close to the scale of the lander. This suggests that future landing site selection efforts using these techniques and methods will be similarly successful.

## 13 Summary and Conclusions

Selection of the InSight landing site took place over 6 years in which targeted remote sensing data were used to characterize the surface and atmosphere for safe entry, descent, and landing. The comprehensive analysis of remote sensing data made predictions about both the atmosphere and surface that are tested herein from data acquired by the spacecraft. We find a close correspondence between the expected atmosphere and surface and those found by InSight. In addition, the expected geologic setting of the surface and shallow subsurface structure inferred from orbital data is consistent with surface geologic and geophysical observations.

General predictions of the landing site before landing were that the landing site in western Elysium Planitia would be safe for the InSight landing system and that the instruments could be deployed onto the surface. Further, the smooth plains upon which the InSight landing site is located would be generally similar to the Mars Exploration Rover Spirit landing site with relatively low rock abundance, low slopes, a moderately dusty surface and be composed of an impact fragmented, relatively fine grained regolith 3–10 m thick underlain by Hesperian to Early Amazonian basaltic lava flows that was modified by eolian and mass wasting processes. InSight landed safely on 26 November 2018, and the instruments were successfully deployed onto the surface in the workspace; all of these predictions are consistent with surface observations.

InSight arrived at Mars about 5 months after a large global dust storm had initiated, by which time global observations of the atmosphere indicated that it had reached expected background conditions. The deceleration‐derived temperature profile and surface pressure compared well with the envelope of modeled temperature profiles (within 1*σ*) and the expected surface pressure. Although the reconstructed density profile is consistently around 1*σ* below the mean model density, the atmosphere encountered by InSight was well within the capabilities of the landing system and the expected model conditions for the background atmosphere.

InSight landed on a surface with moderate thermal inertia and intermediate to relatively high albedo generally similar to the Viking, Spirit, Phoenix, and Curiosity landing sites. Orbital TES and THEMIS thermal inertia is ~230 and ~166 J m^−2^ K^−1^ s^−1/2^, respectively, interpreted to be due to a surface composed predominantly of poorly consolidated sand size particles or a mixture of slightly cohesive soils (cohesions of less than a few kilopascals), some rocks and thermally thin coatings of dust, without a steep inertia contrast within the top few tens of centimeters. The relatively high albedo (~0.24) and DCI argues for a thin coating of dust. Surface RAD diurnal temperature measurements yield an average thermal inertia of ~200 J m^−2^ K^−1^ s^−1/2^, similar to orbital derivations, corresponding to poorly consolidated particles of fine sand (~150 μm). The expectation of a moderately dusty surface similar to the dusty portion of the Gusev cratered plains is consistent with the reddish color of the landscape and the extensive dark spot where dust was removed by the landing thrusters.

Orbital estimates of rock abundance from high‐resolution images and thermal differencing in the landing ellipse indicated a surface with a very few rocks. Surface rock counts indicate rock size‐frequency distributions similar to Phoenix and the Spirit landing sites with 1–4% CFA covered as expected and consistent with fragmentation theory predictions. The observed steep increase in number of rocks less than 4 cm diameter was suggested by interpretations of radar polarization echoes.

Orbital estimates of slopes at 100 to 1 m length scale from MOLA extrapolations, HRSC, CTX, and HiRISE DEMs, photoclinometry, and radar returns all indicated a very smooth landing site comparable to the Opportunity and Phoenix landing sites that are consistent with surface observations. Interpretations of radar data indicated no anomalous returns that would affect the proper functioning of the radar altimeter and velocimeter, both of which worked properly. The load bearing surface that InSight landed on was indicated from both the radar data and the thermophysical properties. Surface alteration from retrorockets is as inferred from studies before landing, and no surface observations indicate any violation of planetary protection requirements. Finally, the density of Corinto secondaries in the landing region and their shape, slopes, and depth/diameter ratio as are as expected from HiRISE images.

Orbital analysis of rocky and non‐RECs argued for a poorly consolidated, relatively fine grained, regolith around 3 m thick at the landing site. Geologic mapping, spectra, the Hesperian to Early Amazonian surface crater age, and fragmentation theory argued that the regolith was generated by repeated impacts on basaltic lava flows and that the surface was modified by eolian and mass wasting processes. This geologic origin and shallow subsurface structure is supported by observations from the lander and by measured slow near‐surface seismic velocities. The origin and evolution of the InSight landing site is thus similar to the Gusev cratered plains at the Spirit landing site as predicted.

## Data Availability

All data from NASA spacecraft are available in the NASA Planetary Data System archive. All InSight and other remote sensing data discussed in this paper are in the Geosciences node (at https://pds-geosciences.wustl.edu/missions/insight/index.htm). All imaging data are in the Cartography and Imaging Node (at https://pds-imaging.jpl.nasa.gov/). Rock data used in Figures 5–10, crater data in Figure 12, and the regolith triangulation results in Figures 16–18 are available in Golombek (2020).

## References

[jgre21429-bib-0001] Anderson, F. S. , Haldemann, A. F. C. , Bridges, N. T. , Golombek, M. P. , Parker, T. J. , & Neumann, G. (2003). Analysis of MOLA data for the Mars Exploration Rover landing sites. Journal of Geophysical Research, 108(E12), 8084 10.1029/2003JE002125

[jgre21429-bib-0002] Arvidson, R. , Adams, D. , Bonfiglio, G. , Christensen, P. , Cull, S. , Golombek, M. , Guinn, J. , Guinness, E. , Heet, T. , Kirk, R. , Knudson, A. , Malin, M. , Mellon, M. , McEwen, A. , Mushkin, A. , Parker, T. , Seelos, F. IV , Seelos, K. , Smith, P. , Spencer, D. , Stein, T. , & Tamppari, L. (2008). Mars Exploration Program 2007 Phoenix landing site selection and characteristics. Journal of Geophysical Research, 113, E00A03 10.1029/2007JE003021

[jgre21429-bib-0003] Arvidson, R. E. , Bonitz, R. G. , Robinson, M. L. , Carsten, J. L. , Volpe, R. A. , Trebi‐Ollennu, A. , Mellon, M. T. , Chu, P. C. , Davis, K. R. , Wilson, J. J. , Shaw, A. S. , Greenberger, R. N. , Siebach, K. L. , Stein, T. C. , Cull, S. C. , Goetz, W. , Morris, R. V. , Ming, D. W. , Keller, H. U. , Lemmon, M. T. , Sizemore, H. G. , & Mehta, M. (2009). Results from the Mars Phoenix Lander Robotic Arm experiment. Journal of Geophysical Research, 114, E00E02 10.1029/2009JE003408

[jgre21429-bib-0004] Banerdt, W. B. , Smrekar, S. E. , Banfield, D. , Giardini, D. , Golombek, M. , Johnson, C. L. , Lognonné, P. , Spiga, A. , Spohn, T. , Perrin, C. , Stähler, S. C. , Antonangeli, D. , Asmar, S. , Beghein, C. , Bowles, N. , Bozdag, E. , Chi, P. , Christensen, U. , Clinton, J. , Collins, G. S. , Daubar, I. , Dehant, V. , Drilleau, M. , Fillingim, M. , Folkner, W. , Garcia, R. F. , Garvin, J. , Grant, J. , Grott, M. , Grygorczuk, J. , Hudson, T. , Irving, J. C. E. , Kargl, G. , Kawamura, T. , Kedar, S. , King, S. , Knapmeyer‐Endrun, B. , Knapmeyer, M. , Lemmon, M. , Lorenz, R. , Maki, J. N. , Margerin, L. , McLennan, S. M. , Michaut, C. , Mimoun, D. , Mittelholz, A. , Mocquet, A. , Morgan, P. , Mueller, N. T. , Murdoch, N. , Nagihara, S. , Newman, C. , Nimmo, F. , Panning, M. , Pike, W. T. , Plesa, A. C. , Rodriguez, S. , Rodriguez‐Manfredi, J. A. , Russell, C. T. , Schmerr, N. , Siegler, M. , Stanley, S. , Stutzmann, E. , Teanby, N. , Tromp, J. , van Driel, M. , Warner, N. , Weber, R. , & Wieczorek, M. (2020). Early results from the InSight Mission: Mission overview and global seismic activity. Nature Geoscience, 13(3), 183–189. 10.1038/s41561-020-0544-y

[jgre21429-bib-0005] Banfield, D. , Rodriguez‐Manfredi, J. A. , Russell, C. T. , Rowe, K. M. , Leneman, D. , Lai, H. R. , Cruce, P. R. , Means, J. D. , Johnson, C. L. , Mittelholz, A. , Joy, S. P. , Chi, P. J. , Mikellides, I. G. , Carpenter, S. , Navarro, S. , Sebastian, E. , Gomez‐Elvira, J. , Torres, J. , Mora, L. , Peinado, V. , Lepinette, A. , Team, T. T. W. I. N. S. , Hurst, K. , Lognonné, P. , Smrekar, S. E. , & Banerdt, W. B. (2019). InSight Auxiliary Payload Sensor Suite (APSS). Space Science Reviews, 215(1), 4 10.1007/s11214-018-0570-x

[jgre21429-bib-0006] Banfield, D. , Spiga, A. , Newman, C. , Forget, F. , Lemon, M. , Lorenz, R. , Murdoch, N. , Viudez‐Moreiras, D. , & Pla‐Garcia, J. (2020). The atmosphere of Mars as observed by InSight. Nature Geoscience, 13(3), 190–198. 10.1038/s41561-020-0534-0

[jgre21429-bib-0007] Bern, M. , & Eppstein, D. (1992). Mesh generation and optimal triangulation In Du D. Z., & Hwang F. (Eds.), Computing in Euclidean geometry, Singapore: World Scientific. (Also, Xerox Palo Alto Research Center Technical report CSL‐92‐1)

[jgre21429-bib-0008] Beyer, R. A. (2017). Meter‐scale slopes of candidate InSight landings sites from point photoclinometry. Space Science Reviews, 211(1‐4), 97–107. 10.1007/s11214-016-0287-7

[jgre21429-bib-0009] Blanchard, R. C. , & Desai, P. N. (2011). Mars Phoenix entry, descent, and landing trajectory and atmosphere reconstruction. Journal of Spacecraft and Rockets, 48(5), 809–822. 10.2514/1.46274

[jgre21429-bib-0010] Bloom, C. , Golombek, M. , Warner, N. , Wigton, N. (2014). Size frequency distribution and ejection velocity of Corinto crater secondaries in Elysium Planitia. Eighth International Conference on Mars, Pasadena, CA, July 14–18, Lunar and Planetary Institute, Houston, Abstract #1289.

[jgre21429-bib-0011] Campbell, B. A. (2001). Radar backscatter from Mars: Properties of rock‐strewn surfaces. Icarus, 150(1), 38–47. 10.1006/icar.2000.6566

[jgre21429-bib-0012] Campbell, B. A. , Putzig, N. E. , Carter, L. M. , Morgan, G. A. , Phillips, R. J. , & Plaut, J. J. (2013). Roughness and near‐surface density of Mars from SHARAD radar echoes. Journal of Geophysical Research: Planets, 118, 436–450. 10.1002/jgre.20050

[jgre21429-bib-0013] Charalambous, C. (2014). On the evolution of particle fragmentation with applications to planetary surfaces. PhD Thesis, Imperial College London.

[jgre21429-bib-0014] Charalambous, C. , Golombek, M. , Pike, T. , Warner, N. H. , Weitz, C. , Ansan, V. , Hauber, E. , Grant, J. , Williams, N. , Wilson, S. , DeMott, A , Kopp, M. , Lethcoe, H. (2019). Rock distributions at the InSight landing site and implications based on fragmentation theory. 50^th^ Lunar and Planetary Science, Abstract #2812, Lunar and Planetary Institute, Houston.

[jgre21429-bib-0015] Chen, A. , Cianciolo, A. , Vasavada, A. R. , Karlgaard, C. , Barnes, J. , Cantor, B. , Kass, D. , Rafkin, S. , & Tyler, D. (2014). Reconstruction of atmospheric properties from the Mars Science Laboratory entry, descent, and landing. Journal of Spacecraft and Rockets, 51(4), 1062–1075. 10.2514/1.A32708

[jgre21429-bib-0016] Christensen, P. R. (1986). The spatial distribution of rocks on Mars. Icarus, 68(2), 217–238. 10.1016/0019-1035(86)90020-5

[jgre21429-bib-0017] Christensen, P. R. , Bandfield, J. L. , Hamilton, V. E. , Ruff, S. W. , Kieffer, H. H. , Titus, T. N. , Malin, M. C. , Morris, R. V. , Lane, M. D. , Clark, R. L. , Jakosky, B. M. , Mellon, M. T. , Pearl, J. C. , Conrath, B. J. , Smith, M. D. , Clancy, R. T. , Kuzmin, R. O. , Roush, T. , Mehall, G. L. , Gorelick, N. , Bender, K. , Murray, K. , Dason, S. , Greene, E. , Silverman, S. , & Greenfield, M. (2001). Mars Global Surveyor Thermal Emission Spectrometer experiment: Investigation description and surface science results. Journal of Geophysical Research, 106(E10), 23,823–23,871. 10.1029/2000JE001370

[jgre21429-bib-0018] Christensen, P. R. , & Moore, H. J. (1992). The Martian surface layer In Kieffer H. H., Jakosky B. M., Snyder C. W., & Matthews M. S. (Eds.), Mars (pp. 686–727). Tucson: University of Arizona Press.

[jgre21429-bib-0019] Craddock, R. A. , & Golombek, M. P. (2016). Characteristics of terrestrial basaltic rock populations: Implications for Mars lander and rover science and safety. Icarus, 274, 50–72. 10.1016/j.icarus.2016.02.042

[jgre21429-bib-0020] Daubar, I. J. , Dundas, C. M. , Byrne, S. , Geissler, P. E. , Bart, G. D. , McEwen, A. S. , Russell, P. S. , Chojnacki, M. , & Golombek, M. P. (2016). Changes in blast zone albedo patterns around new Martian impact craters. Icarus, 267, 86–105. 10.1016/j.icarus.2015.11.032

[jgre21429-bib-0021] Daubar, I. J. , McEwen, A. S. , & Golombek, M. P. (2015). Albedo changes at Martian landing sites. 46^th^ Lunar and Planetary Science, Abstract #2225, Lunar and Planetary Institute, Houston.

[jgre21429-bib-0022] Desai, P. N. , Schoenenberger, M. , & Cheatwood, F. M. (2006). Mars Exploration Rover six‐degree‐of‐freedom entry trajectory analysis. Journal of Spacecraft and Rockets, 43(5), 1019–1025. 10.2514/1.6008

[jgre21429-bib-0023] Fergason, R. , Kirk, R. L. , Cushing, G. , Galuzska, D. M. , Golombek, M. P. , Hare, T. M. , Howington‐Kraus, E. , Kipp, D. M. , & Redding, B. L. (2017). Analysis of local slopes at the InSight landing site on Mars. Space Science Reviews, 211(1‐4), 109–133. 10.1007/s11214-016-0292-x

[jgre21429-bib-0024] Garvin, J. B. , Dotson, R. , Williams, N. , Maki, J. , Deen, R. , Abarca, H. , Ruoff, N. , Trebi‐Ollennu, A. (2019). Microtopography of the Mars InSight landing site: Geological implications. 50^th^ Lunar and Planetary Science Conference, Abstract #1705.

[jgre21429-bib-0025] Garvin, J. B. , Frawley, J. J. , & Abshire, J. B. (1999). Vertical roughness of Mars from Mars Orbiter Laser Altimeter. Geophysical Research Letters, 26(3), 381–384. 10.1029/1998GL900309

[jgre21429-bib-0026] Golombek, M. (2020). Data for “Assessment of InSight landing site predictions” [data set]. CaltechDATA. 10.22002/D1.1422 PMC7507760 32999801

[jgre21429-bib-0027] Golombek, M. , Bloom, C. , Wigton, N. , & Warner, N. (2014). Constraints on the age of Corinto crater from mapping secondaries in Elysium Planitia on Mars. 45th Lunar and Planetary Science, Lunar and Planetary Institute, Houston. Abstract #1470.

[jgre21429-bib-0028] Golombek, M. , Charalambous, C. , Pike, W. T. , & Sullivan, R . (2020). The origin of sand and dust on Mars: Evidence from the InSight landing site. 51^st^ Lunar and Planetary Science, Abstract #2744, Lunar and Planetary Institute, Houston.

[jgre21429-bib-0029] Golombek, M. , Grant, J. , Kipp, D. , Vasavada, A. , Kirk, R. , Fergason, R. , Bellutta, P. , Calef, F. , Larsen, K. , Katayama, Y. , Huertas, A. , Beyer, R. , Chen, A. , Parker, T. , Pollard, B. , Lee, S. , Sun, Y. , Hoover, R. , Sladek, H. , Grotzinger, J. , Welch, R. , Noe Dobrea, E. , Michalski, J. , & Watkins, M. (2012). Selection of the Mars Science Laboratory landing site. Space Science Reviews, 170(1‐4), 641–737. 10.1007/s11214-012-9916-y

[jgre21429-bib-0030] Golombek, M. , Grott, M. , Kargl, G. , Andrade, J. , Marshall, J. , Warner, N. , Teanby, N. A. , Ansan, V. , Hauber, E. , Voigt, J. , Lichtenheldt, R. , Knapmeyer‐Endrun, B. , Daubar, I. J. , Kipp, D. , Muller, N. , Lognonné, P. , Schmelzbach, C. , Banfield, D. , Trebi‐Ollennu, A. , Maki, J. , Kedar, S. , Mimoun, D. , Murdoch, N. , Piqueux, S. , Delage, P. , Pike, W. T. , Charalambous, C. , Lorenz, R. , Fayon, L. , Lucas, A. , Rodriguez, S. , Morgan, P. , Spiga, A. , Panning, M. , Spohn, T. , Smrekar, S. , Gudkova, T. , Garcia, R. , Giardini, D. , Christensen, U. , Nicollier, T. , Sollberger, D. , Robertsson, J. , Ali, K. , Kenda, B. , & Banerdt, W. B. (2018). Geology and physical properties investigations by the InSight lander. Space Science Reviews, 214(5), 84 10.1007/s11214-018-0512-7

[jgre21429-bib-0031] Golombek, M. , Huertas, A. , Kipp, D. , & Calef, F. (2012). Detection and characterization of rocks and rock size‐frequency distributions at the final four Mars Science Laboratory landing sites. Marsyas, 7, 1–22. 10.1555/mars.2012.0001

[jgre21429-bib-0032] Golombek, M. , Kipp, D. , Kipp, D. , Warner, N. , Daubar, I. J. , Fergason, R. , Kirk, R. , Beyer, R. , Huertas, A. , Piqueux, S. , Putzig, N. E. , Campbell, B. A. , Morgan, G. A. , Charalambous, C. , Pike, W. T. , Gwinner, K. , Calef, F. , Kass, D. , Mischna, M. , Ashley, J. , Bloom, C. , Wigton, N. , Hare, T. , Schwartz, C. , Gengl, H. , Redmond, L. , Trautman, M. , Sweeney, J. , Grima, C. , Smith, I. B. , Sklyanskiy, E. , Lisano, M. , Benardini, J. , Smrekar, S. , Lognonné, P. , & Banerdt, W. B. (2017). Selection of the InSight landing site. Space Science Reviews, 211(1‐4), 5–95. 10.1007/s11214-016-0321-9

[jgre21429-bib-0033] Golombek, M. , & Rapp, D. (1997). Size‐frequency distributions of rocks on Mars and Earth analog sites: Implications for future landed missions. Journal of Geophysical Research, 102(E2), 4117–4129. 10.1029/96JE03319

[jgre21429-bib-0034] Golombek, M. , Warner, N. H. , Grant, J. A. , Hauber, E. , Ansan, V. , Weitz, C. M. , Williams, N. , Charalambous, C. , Wilson, S. A. , DeMott, A. , Kopp, M. , Lethcoe‐Wilson, H. , Berger, L. , Hausmann, R. , Marteau, E. , Vrettos, C. , Trussell, A. , Folkner, W. , le Maistre, S. , Mueller, N. , Grott, M. , Spohn, T. , Piqueux, S. , Millour, E. , Forget, F. , Daubar, I. , Murdoch, N. , Lognonné, P. , Perrin, C. , Rodriguez, S. , Pike, W. T. , Parker, T. , Maki, J. , Abarca, H. , Deen, R. , Hall, J. , Andres, P. , Ruoff, N. , Calef, F. , Smrekar, S. , Baker, M. M. , Banks, M. , Spiga, A. , Banfield, D. , Garvin, J. , Newman, C. E. , & Banerdt, W. B. (2020). Geology of the InSight landing site on Mars. Nature Communications, 11(1), 1014 10.1038/s4167-020-14679-1 PMC7039939 32094337

[jgre21429-bib-0035] Golombek, M. P. , Arvidson, R. E. , Bell, J. F. III , Christensen, P. R. , Crisp, J. A. , Crumpler, L. S. , Ehlmann, B. L. , Fergason, R. L. , Grant, J. A. , Greeley, R. , Haldemann, A. F. C. , Kass, D. M. , Parker, T. J. , Schofield, J. T. , Squyres, S. W. , & Zurek, R. W. (2005). Assessment of Mars Exploration Rover landing site predictions. Nature, 436(7047), 44–48. 10.1038/nature03600 16001058

[jgre21429-bib-0036] Golombek, M. P. , Charalambous, C. , Pike, W. T. , & Sullivan, R . (2018). The origin of sand on Mars. 49^th^ Lunar and Planetary Science, Abstract #2319, Lunar and Planetary Institute, Houston.

[jgre21429-bib-0037] Golombek, M. P. , Cook, R. A. , Moore, H. J. , & Parker, T. J. (1997). Selection of the Mars Pathfinder landing site. Journal of Geophysical Research, 102(E2), 3967–3988. 10.1029/96JE03318

[jgre21429-bib-0038] Golombek, M. P. , Crumpler, L. S. , Grant, J. A. , Greeley, R. , Cabrol, N. A. , Parker, T. J. , Rice, J. W. Jr. , Ward, J. G. , Arvidson, R. E. , Moersch, J. E. , Fergason, R. L. , Christensen, P. R. , Castaño, A. , Castaño, R. , Haldemann, A. F. C. , Li, R. , Bell, J. F. III , & Squyres, S. W. (2006). Geology of the Gusev cratered plains from the Spirit rover traverse. Journal of Geophysical Research, 111, E02S07 10.1029/2005JE002503

[jgre21429-bib-0039] Golombek, M. P. , Grant, J. A. , Crumpler, L. S. , Greeley, R. , Arvidson, R. E. , Bell, J. F. III , Weitz, C. M. , Sullivan, R. , Christensen, P. R. , Soderblom, L. A. , & Squyres, S. W. (2006). Erosion rates at the Mars Exploration Rover landing sites and long‐term climate change on Mars. Journal of Geophysical Research, 111, E12S10 10.1029/2006JE002754

[jgre21429-bib-0040] Golombek, M. P. , Grant, J. A. , Parker, T. J. , Kass, D. M. , Crisp, J. A. , Squyres, S. W. , Haldemann, A. F. C. , Adler, M. , Lee, W. J. , Bridges, N. T. , Arvidson, R. E. , Carr, M. H. , Kirk, R. L. , Knocke, P. C. , Roncoli, R. B. , Weitz, C. M. , Schofield, J. T. , Zurek, R. W. , Christensen, P. R. , Fergason, R. L. , Anderson, F. S. , & Rice, J. W. Jr. (2003). Selection of the Mars Exploration Rover landing sites. Journal of Geophysical Research, 108(E12), 8072 10.1029/2003JE002074

[jgre21429-bib-0041] Golombek, M. P. , Haldemann, A. F. C. , Forsberg‐Taylor, N. K. , DiMaggio, E. N. , Schroeder, R. D. , Jakosky, B. M. , Mellon, M. T. , & Matijevic, J. R. (2003). Rock size‐frequency distributions on Mars and implications for Mars Exploration Rover landing safety and operations. Journal of Geophysical Research, 108(E12), 8086 10.1029/2002JE002035

[jgre21429-bib-0042] Golombek, M. P. , Haldemann, A. F. C. , Simpson, R. A. , Fergason, R. L. , Putzig, N. E. , Arvidson, R. E. , Bell, J. F. III , & Mellon, M. T. (2008). Martian surface properties from joint analysis of orbital, Earth‐based, and surface observations In Bell J. F. III (Ed.), The Martian surface: Composition, mineralogy and physical properties (pp. 468–497). Cambridge: Cambridge University Press. Chap. 21

[jgre21429-bib-0043] Golombek, M. P. , Huertas, A. , Marlow, J. , McGrane, B. , Klein, C. , Martinez, M. , Arvidson, R. E. , Heet, T. , Barry, L. , Seelos, K. , Adams, D. , Li, W. , Matijevic, J. R. , Parker, T. , Sizemore, H. G. , Mellon, M. , McEwen, A. S. , Tamppari, L. K. , & Cheng, Y. (2008). Size‐frequency distributions of rocks on the northern plains of Mars with special reference to Phoenix landing surfaces. Journal of Geophysical Research, 113, E00A09 10.1029/2007JE003065

[jgre21429-bib-0044] Golombek, M. P. , Moore, H. J. , Haldemann, A. F. C. , Parker, T. J. , & Schofield, J. T. (1999). Assessment of Mars Pathfinder landing site predictions. Journal of Geophysical Research, 104(E4), 8585–8594. 10.1029/1998JE900015

[jgre21429-bib-0045] Golombek, M. P. , & Phillips, R. J. (2010). Mars tectonics In Watters T. R., & Schultz R. A. (Eds.), Planetary tectonics (pp. 183–232). Cambridge: Cambridge University Press. Chap. 5

[jgre21429-bib-0046] Golombek, M. P. , Warner, N. H. , Ganti, V. , Lamb, M. P. , Parker, T. J. , Fergason, R. L. , & Sullivan, R. (2014). Small crater modification on Meridiani Planum and implications for erosion rates and climate change on Mars. Journal of Geophysical Research: Planets, 119, 2522–2547. 10.1002/2014JE004658

[jgre21429-bib-0047] Gómez‐Elvira, J. , Armiens, C. , Castaner, L. , Domínguez, M. , Genzer, M. , Gómez, F. , Haberle, R. , Harri, A.‐M. , Jiménez, V. , Kahanpää, H. , Kowalski, L. , Lepinette, A. , Martín, J. , Martínez‐Frías, J. , McEwan, I. , Mora, L. , Moreno, J. , Navarro, S. , de Pablo, M. A. , Peinado, V. , Peña, A. , Polkko, J. , Ramos, M. , Renno, N. O. , Ricart, J. , Richardson, M. , Rodríguez‐Manfredi, J. , Romeral, J. , Sebastián, E. , Serrano, J. , de la Torre Juárez, M. , Torres, J. , Torrero, F. , Urquí, R. , Vázquez, L. , Velasco, T. , Verdasca, J. , Zorzano, M.‐P. , & Martín‐Torres, J. (2012). REMS: The environmental sensor suite for the Mars Science Laboratory Rover. Space Science Reviews, 170(1‐4), 583–640. 10.1007/s11214-012-9921-1

[jgre21429-bib-0048] Grant, J. A. , Arvidson, R. , Bell, J. F. III , Cabrol, N. A. , Carr, M. H. , Christensen, P. , Crumpler, L. , Marais, D. J. D. , Ehlmann, B. L. , Farmer, J. , Golombek, M. , Grant, F. D. , Greeley, R. , Herkenhoff, K. , Li, R. , McSween, H. Y. , Ming, D. W. , Moersch, J. , Rice, J. W. Jr. , Ruff, S. , Richter, L. , Squyres, S. , Sullivan, R. , & Weitz, C. (2004). Surficial deposits at Gusev crater along Spirit rover traverses. Science, 305(5685), 807–810. 10.1126/science.1099849 15297659

[jgre21429-bib-0049] Grant, J. A. , Arvidson, R. E. , Crumpler, L. S. , Golombek, M. P. , Hahn, B. , Haldemann, A. F. C. , Li, R. , Soderblom, L. A. , Squyres, S. W. , Wright, S. P. , & Watters, W. A. (2006). Crater gradation in Gusev crater and Meridiani Planum, Mars. Journal of Geophysical Research, 111, E02S08 10.1029/2005JE002465

[jgre21429-bib-0050] Grant, J. A. , Warner, N. H. , Weitz, C. M. , Golombek, M. P. , Wilson, S. A. , Baker, M. , Hauber, E. , Ansan, V. , Charalambous, C. , Williams, N. , Calef, F. , Pike, W. T. , DeMott, A. , Kopp, M. , Lethcoe, H. , & Banks, M. E. (2020). Degradation of Homestead hollow at the InSight landing site based on the distribution and properties of local deposits. Journal of Geophysical Research; Planets, 125, e2019JE006350 10.1029/2019JE006350

[jgre21429-bib-0051] Grant, J. A. , Wilson, S. A. , Ruff, S. W. , Golombek, M. P. , & Koestler, D. L. (2006). Distribution of rocks on the Gusev Plains and on Husband Hill, Mars. Geophysical Research Letters, 33, L16202 10.1029/2006GL026964

[jgre21429-bib-0052] Greeley, R. , Foing, B. H. , McSween, H. Y. Jr. , Neukum, G. , Pinet, P. , van Kan, M. , Werner, S. C. , Williams, D. A. , & Zegers, T. E. (2005). Fluid lava flows in Gusev crater. Mars. Journal of Geophysical Research, 110, E05008 10.1029/2005JE002401

[jgre21429-bib-0053] Grover, R. M. , Cichy, B. D. , & Desai, P. N. (2011). Overview of the Phoenix entry, descent, and landing system architecture. Journal of Spacecraft and Rockets, 48(5), 706–712. 10.2514/1.46548

[jgre21429-bib-0054] Guzewich, S. D. , Lemmon, M. , Smith, C. L. , Martínez, G. , de Vicente‐Retortillo, Á. , Newman, C. E. , Baker, M. , Campbell, C. , Cooper, B. , Gómez‐Elvira, J. , Harri, A.‐. M. , Hassler, D. , Martin‐Torres, F. J. , McConnochie, T. , Moores, J. E. , Kahanpää, H. , Khayat, A. , Richardson, M. I. , Smith, M. D. , Sullivan, R. , de la Torre Juarez, M. , Vasavada, A. R. , Viúdez‐Moreiras, D. , Zeitlin, C. , & Mier, M.‐. P. Z. (2019). Mars Science Laboratory observations of the 2018/Mars year 34 global dust storm. Geophysical Research Letters, 46, 71–79. 10.1029/2018GL080839

[jgre21429-bib-0055] Gwinner, K. , Jaumann, R. , Hauber, E. , Hoffmann, H. , Heipke, C. , Oberst, J. , Neukum, G. , Ansan, V. , Bostelmann, J. , Dumke, A. , Elgner, S. , Erkeling, G. , Fueten, F. , Hiesinger, H. , Hoekzema, N. M. , Kersten, E. , Loizeau, D. , Matz, K. D. , McGuire, P. C. , Mertens, V. , Michael, G. , Pasewaldt, A. , Pinet, P. , Preusker, F. , Reiss, D. , Roatsch, T. , Schmidt, R. , Scholten, F. , Spiegel, M. , Stesky, R. , Tirsch, D. , van Gasselt, S. , Walter, S. , Wählisch, M. , & Willner, K. (2016). The High‐Resolution Stereo Camera (HRSC) of Mars Express and its approach to science analysis and mapping for Mars and its satellites. Planetary and Space Science, 126, 93–138. 10.1016/j.pss.2016.02.014

[jgre21429-bib-0056] Harmon, J. K. , Nolan, M. C. , Husmann, D. I. , & Campbell, B. A. (2012). Arecibo radar imagery of Mars: The major volcanic provinces. Icarus, 220(2), 990–1030. 10.1016/j.icarus.2012.06.030

[jgre21429-bib-0057] Hartmann, W. K. (1984). Does crater “saturation equilibrium” occur in the solar system? Icarus, 60(1), 56–74. 10.1016/0019-1035(84)90138-6

[jgre21429-bib-0058] Heet, T. L. , Arvidson, R. E. , Cull, S. C. , Mellon, M. T. , & Seelos, K. D. (2009). Geomorphic and geologic settings of the Phoenix Lander mission landing site. Journal of Geophysical Research, 114, E00E04 10.1029/2009JE003416

[jgre21429-bib-0059] Hudson, T. L. , Deen, R. , Marteau, E. , Golombek, M. , Hurst, K. , Spohn, T. , Grott , Krause, C. , & Knollenberg, J. (2020). InSight HP^3^ mole near‐surface motion and subsurface implications. 51^st^ Lunar and Planetary Science, Abstract #1217, Lunar and Planetary Institute, Houston.

[jgre21429-bib-0060] Hundal, C. B. , Golombek, M. P. , & Daubar, I. J. (2017). Chronology of fresh rayed craters in Elysium Planitia, Mars. 48^th^ Lunar and Planetary Science, Abstract #1726, Lunar and Planetary Institute, Houston.

[jgre21429-bib-0061] Kahre, M. A. , Murphy, J. R. , Newman, C. E. , Wilson, R. J. , Cantor, B. A. , Lemmon, M. T. , & Wolff, M. J. (2017). The Mars dust cycle In Haberle R. H., Clancy R. T., Forget F., Smith M. D., & Zurek R. W. (Eds.), The atmosphere and climate of Mars (chap. 10, pp. 295–337). Cambridge, UK: Cambridge University Press 10.1017/9781129060172

[jgre21429-bib-0062] Karlgaard, C. D. , Korzun, A. M. , Schoenenberger, M. , Bonfiglio, E. P. , Kass, D. M. , & Grover, M. R. (2020). Mars InSight entry, descent, and landing trajectory and atmosphere reconstruction. AIAA Scitech 2020 Forum, 6‐10 January 2020, Orlando, FL, 10.2514/6.2020-1271

[jgre21429-bib-0063] Kass, D. M. , Kleinböhl, A. , McCleese, D. J. , Schofield, J. T. , & Smith, M. D. (2016). Interannual similarity in the Martian atmosphere during the dust storm season. Geophysical Research Letters, 43, 6111–6118. 10.1002/2016GL068978

[jgre21429-bib-0064] Kass, D. M. , Schofield, J. T. , Kleinböhl, A. , McCleese, D. J. , Heavens, N. G. , Shirley, J. H. , & Steele, L. (2020). Mars Climate Sounder observations of Mars' 2018 global dust event. Geophysical Research Letters, 42, 2019GL083931 10.1029/2019GL083931

[jgre21429-bib-0065] Lognonné, P. , Banerdt, W. B. , Giardini, D. , Pike, W. T. , Christensen, U. , Laudet, P. , de Raucourt, S. , Zweifel, P. , Calcutt, S. , Bierwirth, M. , Hurst, K. J. , Ijpelaan, F. , Umland, J. W. , Llorca‐Cejudo, R. , Larson, S. A. , Garcia, R. F. , Kedar, S. , Knapmeyer‐Endrun, B. , Mimoun, D. , Mocquet, A. , Panning, M. P. , Weber, R. C. , Sylvestre‐Baron, A. , Pont, G. , Verdier, N. , Kerjean, L. , Facto, L. J. , Gharakanian, V. , Feldman, J. E. , Hoffman, T. L. , Klein, D. B. , Klein, K. , Onufer, N. P. , Paredes‐Garcia, J. , Petkov, M. P. , Willis, J. R. , Smrekar, S. E. , Drilleau, M. , Gabsi, T. , Nebut, T. , Robert, O. , Tillier, S. , Moreau, C. , Parise, M. , Aveni, G. , Ben Charef, S. , Bennour, Y. , Camus, T. , Dandonneau, P. A. , Desfoux, C. , Lecomte, B. , Pot, O. , Revuz, P. , Mance, D. , tenPierick, J. , Bowles, N. E. , Charalambous, C. , Delahunty, A. K. , Hurley, J. , Irshad, R. , Liu, H. , Mukherjee, A. G. , Standley, I. M. , Stott, A. E. , Temple, J. , Warren, T. , Eberhardt, M. , Kramer, A. , Kühne, W. , Miettinen, E. P. , Monecke, M. , Aicardi, C. , André, M. , Baroukh, J. , Borrien, A. , Bouisset, A. , Boutte, P. , Brethomé, K. , Brysbaert, C. , Carlier, T. , Deleuze, M. , Desmarres, J. M. , Dilhan, D. , Doucet, C. , Faye, D. , Faye‐Refalo, N. , Gonzalez, R. , Imbert, C. , Larigauderie, C. , Locatelli, E. , Luno, L. , Meyer, J. R. , Mialhe, F. , Mouret, J. M. , Nonon, M. , Pahn, Y. , Paillet, A. , Pasquier, P. , Perez, G. , Perez, R. , Perrin, L. , Pouilloux, B. , Rosak, A. , Savin de Larclause, I. , Sicre, J. , Sodki, M. , Toulemont, N. , Vella, B. , Yana, C. , Alibay, F. , Avalos, O. M. , Balzer, M. A. , Bhandari, P. , Blanco, E. , Bone, B. D. , Bousman, J. C. , Bruneau, P. , Calef, F. J. , Calvet, R. J. , D'Agostino, S. A. , de los Santos, G. , Deen, R. G. , Denise, R. W. , Ervin, J. , Ferraro, N. W. , Gengl, H. E. , Grinblat, F. , Hernandez, D. , Hetzel, M. , Johnson, M. E. , Khachikyan, L. , Lin, J. Y. , Madzunkov, S. M. , Marshall, S. L. , Mikellides, I. G. , Miller, E. A. , Raff, W. , Singer, J. E. , Sunday, C. M. , Villalvazo, J. F. , Wallace, M. C. , Banfield, D. , Rodriguez‐Manfredi, J. A. , Russell, C. T. , Trebi‐Ollennu, A. , Maki, J. N. , Beucler, E. , Böse, M. , Bonjour, C. , Berenguer, J. L. , Ceylan, S. , Clinton, J. , Conejero, V. , Daubar, I. , Dehant, V. , Delage, P. , Euchner, F. , Estève, I. , Fayon, L. , Ferraioli, L. , Johnson, C. L. , Gagnepain‐Beyneix, J. , Golombek, M. , Khan, A. , Kawamura, T. , Kenda, B. , Labrot, P. , Murdoch, N. , Pardo, C. , Perrin, C. , Pou, L. , Sauron, A. , Savoie, D. , Stähler, S. , Stutzmann, E. , Teanby, N. A. , Tromp, J. , van Driel, M. , Wieczorek, M. , Widmer‐Schnidrig, R. , & Wookey, J. (2019). SEIS: Insight's Seismic Experiment for Internal Structure of Mars. Space Science Reviews, 215(1), 12 10.1007/s11214-018-0574-6 30880848 PMC6394762

[jgre21429-bib-0066] Lognonné, P. , Banerdt, W. B. , Pike, W. T. , Giardini, D. , Christensen, U. , Garcia, R. F. , Kawamura, T. , Kedar, S. , Knapmeyer‐Endrun, B. , Margerin, L. , Nimmo, F. , Panning, M. , Tauzin, B. , Scholz, J. R. , Antonangeli, D. , Barkaoui, S. , Beucler, E. , Bissig, F. , Brinkman, N. , Calvet, M. , Ceylan, S. , Charalambous, C. , Davis, P. , van Driel, M. , Drilleau, M. , Fayon, L. , Joshi, R. , Kenda, B. , Khan, A. , Knapmeyer, M. , Lekic, V. , McClean, J. , Mimoun, D. , Murdoch, N. , Pan, L. , Perrin, C. , Pinot, B. , Pou, L. , Menina, S. , Rodriguez, S. , Schmelzbach, C. , Schmerr, N. , Sollberger, D. , Spiga, A. , Stähler, S. , Stott, A. , Stutzmann, E. , Tharimena, S. , Widmer‐Schnidrig, R. , Andersson, F. , Ansan, V. , Beghein, C. , Böse, M. , Bozdag, E. , Clinton, J. , Daubar, I. , Delage, P. , Fuji, N. , Golombek, M. , Grott, M. , Horleston, A. , Hurst, K. , Irving, J. , Jacob, A. , Knollenberg, J. , Krasner, S. , Krause, C. , Lorenz, R. , Michaut, C. , Myhill, R. , Nissen‐Meyer, T. , ten Pierick, J. , Plesa, A. C. , Quantin‐Nataf, C. , Robertsson, J. , Rochas, L. , Schimmel, M. , Smrekar, S. , Spohn, T. , Teanby, N. , Tromp, J. , Vallade, J. , Verdier, N. , Vrettos, C. , Weber, R. , Banfield, D. , Barrett, E. , Bierwirth, M. , Calcutt, S. , Compaire, N. , Johnson, C. L. , Mance, D. , Euchner, F. , Kerjean, L. , Mainsant, G. , Mocquet, A. , Rodriguez Manfredi, J. A. , Pont, G. , Laudet, P. , Nebut, T. , de Raucourt, S. , Robert, O. , Russell, C. T. , Sylvestre‐Baron, A. , Tillier, S. , Warren, T. , Wieczorek, M. , Yana, C. , & Zweifel, P. (2020). Initial results from SEIS with a focus on shallow Mars structure. Nature Geoscience, 13(3), 213–220. 10.1038/s41561-020-0536-y

[jgre21429-bib-0067] Maddock, R. W. , Dwyer‐Cianciolo, A. M. , Karlgaard, C. D. , Korzun, A. M. , Litton, D. & Zumwalt, C. H. (2020). InSight entry, descent and landing post‐ flight performance assessment. AIAA Scitech 2020 Forum, 6‐10 January 2020, Orlando, FL, 10.2514/6.2020-1270

[jgre21429-bib-0068] Magalhaes, J. A. , Schofield, J. T. , & Seiff, A. (1999). Results of the Mars Pathfinder atmospheric structure investigation. Journal of Geophysical Research, 104(E4), 8943–8955. 10.1029/1998JE900041

[jgre21429-bib-0069] Maki, J. N. , Golombek, M. , Deen, R. , Abaraca, H. , Sorice, C. , Goodsall, T. , Lemmon, M. , Trebi‐Ollennu, A. , & Banerdt, W. B. (2018). The color cameras on the InSight lander. Space Science Reviews, 214, 105 10.1007/s11214-018-0536-z

[jgre21429-bib-0070] Malin, M. C. , Bell, J. F. III , Calvin, W. , Clancy, R. T. , Haberle, R. M. , James, P. B. , Lee, S. W. , Thomas, P. C. , & Caplinger, M. A. (2001). Mars Color Imager (MARCI) on the Mars Climate Orbiter. Journal of Geophysical Research, 106(E8), 17,651–17,672. 10.1029/1999JE001145

[jgre21429-bib-0071] Martin, L. J. , & Zurek, R. W. (1993). An analysis of the history of dust activity on Mars. Journal of Geophysical Research, 98(E2), 3221–3246. 10.1029/92JE02937

[jgre21429-bib-0072] McCleese, D. J. , Schofield, J. T. , Taylor, F. W. , Calcutt, S. B. , Foote, M. C. , Kass, D. M. , Leovy, C. B. , Paige, D. A. , Read, P. L. , & Zurek, R. W. (2007). Mars Climate Sounder: An investigation of thermal and water vapor structure, dust and condensate distributions in the atmosphere, and energy balance of the polar regions. Journal of Geophysical Research, 112, E05S06 10.1029/2006JE002790

[jgre21429-bib-0073] McEwen, A. S. , Eliason, E. M. , Bergstrom, J. W. , Bridges, N. T. , Hansen, C. J. , Delamere, W. A. , Grant, J. A. , Gulick, V. C. , Herkenhoff, K. E. , Keszthelyi, L. , Kirk, R. L. , Mellon, M. T. , Squyres, S. W. , Thomas, N. , & Weitz, C. M. (2007). Mars Reconnaissance Orbiter's High‐Resolution Imaging Science Experiment (HiRISE). Journal of Geophysical Research, 112, E05S02 10.1029/2005JE002605

[jgre21429-bib-0074] McKim, R. J. (1999). Telescopic Martian dust storms: A narrative and catalogue. Memoirs of the British Astronomical Association, 44, 165.

[jgre21429-bib-0075] McSween, H. Y. , Wyatt, M. B. , Gellert, R. , Bell, J. F. III , Morris, R. V. , Herkenhoff, K. E. , Crumpler, L. S. , Milam, K. A. , Stockstill, K. R. , Tornabene, L. L. , Arvidson, R. E. , Bartlett, P. , Blaney, D. , Cabrol, N. A. , Christensen, P. R. , Clark, B. C. , Crisp, J. A. , Des Marais, D. J. , Economou, T. , Farmer, J. D. , Farrand, W. , Ghosh, A. , Golombek, M. , Gorevan, S. , Greeley, R. , Hamilton, V. E. , Johnson, J. R. , Joliff, B. L. , Klingelhöfer, G. , Knudson, A. T. , McLennan, S. , Ming, D. , Moersch, J. E. , Rieder, R. , Ruff, S. W. , Schröder, C. , de Souza, P. A. Jr. , Squyres, S. W. , Wänke, H. , Wang, A. , Yen, A. , & Zipfel, J. (2006). Characterization and petrologic interpretation of olivine‐rich basalts at Gusev crater. Mars. Journal of Geophysical Research, 111, E02S10 10.1029/2005JE002477

[jgre21429-bib-0076] Mehta, M. , Renno, N. O. , Marshall, J. , Grover, M. R. , Sengupta, A. , Rusche, N. A. , Kokab, J. F. , Arvidson, R. E. , Markiewicz, W. J. , Lemmon, M. T. , & Smith, P. H. (2011). Explosive erosion during the Phoenix landing exposes subsurface water on Mars. Icarus, 211(1), 172–194. 10.1016/j.icarus.2010.10.003

[jgre21429-bib-0077] Mehta, M. , Sengupta, A. , Rennó, N. O. , Van Norman, J. W. , Huseman, P. G. , Gulick, D. S. , & Pokora, M. (2013). Thruster plume surface interactions: Applications for spacecraft landings on planetary bodies. AIAA Journal, 51(12), 2800–2818. 10.2514/1.J052408

[jgre21429-bib-0078] Mellon, M. T. , Fergason, R. L. , & Putzig, N. E. (2008). The thermal inertia of the surface of Mars In Bell J. F. III (Ed.), The Martian surface: Composition, mineralogy and physical properties (pp. 399–427). Cambridge: Cambridge University Press. Chap. 19

[jgre21429-bib-0079] Melosh, H. J. (1989). Impact craters: A geologic process. London: Oxford University Press.

[jgre21429-bib-0080] Mueller, K. , & Golombek, M. P. (2004). Compressional structures on Mars. Annual Review of Earth and Planetary Sciences, 32(1), 435–464. 10.1146/annurev.earth.32.101802.120553

[jgre21429-bib-0081] Mueller, N.T. , Grott, M. , Piqueux, S. , Lemmon, M . (2020). Mars soil properties from Phobos eclipse observtions by InSight HP^3^ RAD. 51^st^ Lunar and Planetary Science, Abstract #2150, Lunar and Planetary Institute, Houston.

[jgre21429-bib-0082] Mueller, N. T. , Grott, M. , Piqueux, S. , Spohn, T. , Smrekar, S. E. , Knollenberg, J. , Hudson, T. L. , Spiga, A. , Forget, F. , Millour, E. , Lemmon, M. , Maki, J. , Golombek, M. , & Banerdt, W. B. (2019). The HP^3^ radiometer on InSight. Ninth International Conference on Mars, Pasadena, California ‐ July 22–25, 2019, Abstract #6194, Lunar and Planetary Institute, Houston.

[jgre21429-bib-0083] Neumann, G. A. , Abshire, J. B. , Aharonson, O. , Garvin, J. B. , Sun, X. , & Zuber, M. T. (2003). Mars Orbiter Laser Altimeter pulse width measurements and footprint scale roughness. Geophysical Research Letters, 30(11), 1561 10.1029/2003GL017048

[jgre21429-bib-0084] Nowicki, S. A. , & Christensen, P. R. (2007). Rock abundance on Mars from the Thermal Emission Spectrometer. Journal of Geophysical Research, 112, E05007 10.1029/2006JE002798

[jgre21429-bib-0085] Pan, L. , & Quantin, C. (2018). Regional geological context of the InSight Landing Site from mineralogy and stratigraphy. 49th Lunar and Planetary Science, Lunar and Planetary Institute, Houston, Abstract #1918.

[jgre21429-bib-0086] Pan, L. , Quantin‐Nataf, C. , Tauzin, B. , Michaut, C. , Golombek, M. , Lognonné, P. , Grindrod, P. , Langlais, B. , Gudkova, T. , Stepanova, I. , Rodriguez, S. , & Lucas, A. (2020). Crust stratigraphy and heterogeneities of the first kilometers at the dichotomy boundary in western Elysium Planitia and implications for InSight lander. Icarus, 338, 113511 10.1016/j.icarus.2019.113511

[jgre21429-bib-0087] Parker, T. J. , Golombek, M. P. , Calef, F. J. , Williams, N. R. , LeMaistre, S. , Folkner, W. , Daubar, I. J. , Kipp, D. , Sklyanskiy, E. , Lethcoe‐Wilson, H. , & Hausmann, R . (2019). Localization of the InSight lander. 50^th^ Lunar and Planetary Science, Abstract #1948, Lunar and Planetary Institute, Houston.

[jgre21429-bib-0088] Piqueux, S. , Müller, N. , Grott, M. , Knollenberg, J. , Siegler, M. , Millour, E. , Forget, F. , Lemmon, M. , Golombek, M. , Williams, N. , Maki, J. , Grant, J. , Warner, N. , Ansan, V. , Daubar, I. , Spohn, T. , Smrekar, S. , & Banerdt, B. (2020). Regolith properties near the InSight lander derived from 100 sols of radiometer measurements. 51^st^ Lunar and Planetary Science, Abstract #1309, Lunar and Planetary Institute, Houston.

[jgre21429-bib-0089] Plemmons, D. H. , Mehta, M. , Clark, B. C. , Kounaves, S. P. , Peach, L. L. Jr. , Renno, N. O. , Tamppari, L. , & Young, S. M. M. (2008). Effects of the Phoenix Lander descent thruster plume on the Martian surface. Journal of Geophysical Research, 113, E00A11 10.1029/2007JE003059

[jgre21429-bib-0090] Pleskot, L. K. , & Miner, E. D. (1981). Time variability of Martian bolometric albedo. Icarus, 45(1), 179–201. 10.1016/0019-1035(81)90013-0

[jgre21429-bib-0091] Prince, J. L. , Desai, P. N. , Queen, E. M. , & Grover, M. R. (2011). Mars Phoenix entry, descent, and landing simulation design and modeling analysis. Journal of Spacecraft and Rockets, 48(5), 756–764. 10.2514/1.46561

[jgre21429-bib-0092] Putzig, N. E. , & Mellon, M. T. (2007). Apparent thermal inertia and the surface heterogeneity of Mars. Icarus, 191(1), 68–94. 10.1016/j.icarus.2007.1005.1013

[jgre21429-bib-0093] Putzig, N. E. , Mellon, M. T. , Arvidson, R. E. , & Kretke, K. A. (2005). Global thermal inertia and surface properties of Mars from the MGS mapping mission. Icarus, 173(2), 325–341. 10.1016/j.icarus.2004.08.017

[jgre21429-bib-0094] Putzig, N. E. , Morgan, G. A. , Campbell, B. A. , Grima, C. , Smith, I. B. , Phillips, R. J. , & Golombek, M. P. (2017). Radar‐derived properties of the InSight landing site in western Elysium Planitia on Mars. Space Science Reviews, 211(1‐4), 135–146. 10.1007/s11214-016-0322-8

[jgre21429-bib-0095] Rogers, A. D. , Warner, N. H. , Golombek, M. P. , Head, J. W. III , & Cowart, J. C. (2018). Areally extensive surface bedrock exposures on Mars: Many are clastic rocks, not lavas. Geophysical Research Letters, 45, 1767–1777. 10.1002/2018GL077030 30598561 PMC6310033

[jgre21429-bib-0096] Ruff, S. , & Christensen, P. R. (2002). Bright and dark regions on Mars: Particle size and mineralogical characteristics based on Thermal Emission Spectrometer data. Journal of Geophysical Research, 107(E12), 5127 10.1029/2001JE001580

[jgre21429-bib-0097] Rummel, J. D. , Beaty, D. W. , Jones, M. A. , Bakermans, C. , Barlow, N. G. , Boston, P. J. , Chevrier, V. F. , Clark, B. C. , de Vera, J. P. P. , Gough, R. V. , Hallsworth, J. E. , Head, J. W. , Hipkin, V. J. , Kieft, T. L. , McEwen, A. S. , Mellon, M. T. , Mikucki, J. A. , Nicholson, W. L. , Omelon, C. R. , Peterson, R. , Roden, E. E. , Sherwood Lollar, B. , Tanaka, K. L. , Viola, D. , & Wray, J. J. (2014). A new analysis of Mars “special regions”: Findings of the second MEPAG special regions science analysis group (SR‐SAG2). Astrobiology, 14(11), 887–968. 10.1089/ast.2014.1227 25401393

[jgre21429-bib-0098] Shirley, J. H. , Newman, C. E. , Mischna, M. A. , & Richardson, M. I. (2019). Replication of the historic record of Martian global dust storm occurrence in an atmospheric general circulation model. Icarus, 317, 197–208. 10.1016/j.icarus.2018.07.024

[jgre21429-bib-0099] Smith, D. E. , Zuber, M. T. , Frey, H. V. , Garvin, J. B. , Head, J. W. , Muhleman, D. O. , Pettengill, G. H. , Phillips, R. J. , Solomon, S. C. , Zwally, H. J. , Banerdt, W. B. , Duxbury, T. C. , Golombek, M. P. , Lemoine, F. G. , Neumann, G. A. , Rowlands, D. D. , Aharonson, O. , Ford, P. G. , Ivanov, A. B. , Johnson, C. L. , McGovern, P. J. , Abshire, J. B. , Afzal, R. S. , & Sun, X. (2001). Mars Orbiter Laser Altimeter (MOLA): Experiment summary after the first year of global mapping of Mars. Journal of Geophysical Research, 106(E10), 23,689–23,722. 10.1029/2000JE001364

[jgre21429-bib-0100] Smith, M. D. , Bougher, S. W. , Encrenaz, T. , Forget, F. , & Kleinböhl, A. (2017). Thermal structure and composition In Haberle R. H., Clancy R. T., Forget F., Smith M. D., & Zurek R. W. (Eds.), The atmosphere and climate of Mars (Chap. 4, pp. 42–75). Cambridge, UK: Cambridge University Press 10.1017/9781129060172

[jgre21429-bib-0101] Smith, M. D. , Conrath, R. J. , Pearl, J. C. , & Christensen, P. R. (2002). Thermal Emission Spectrometer observations of Martian planet‐encircling dust storm 2001. Icarus, 157(1), 259–263. 10.1006/icar.2001.6797

[jgre21429-bib-0102] Spohn, T. , Grott, M. , Smrekar, S. E. , Knollenberg, J. , Hudson, T. L. , Mueller, N. , Jänchen, J. , Börner, A. , Wippermann, T. , Krömer, O. , Lichtenheldt, R. , Wisniewski, L. , Grygorczuk, J. , Fittock, M. , Rheershemius, S. , Spröwitz, T. , Kopp, E. , Walter, I. , Plesa, A. C. , Breuer, D. , Morgan, P. , & Banerdt, W. B. (2018). The Heat Flow and Physical Properties Package (HP^3^) for the InSight Mission. Space Science Reviews, 214(5), 96 10.1007/s11214-018-0531-4

[jgre21429-bib-0103] Sweeney, J. , Warner, N. H. , Ganti, V. , Golombek, M. P. , Lamb, M. P. , Fergason, R. , & Kirk, R. (2018). Degradation of 100‐m‐scale impact craters at the InSight landing site on Mars with implications for surface processes and erosion rates in the Hesperian and Amazonian. Journal of Geophysical Research: Planets, 123, 2732–2759. 10.1029/2018JE005618

[jgre21429-bib-0104] Trebi‐Ollennu, A. , Kim, W. , Ali, K. , Khan, O. , Sorice, C. , Bailey, P. , Umland, J. , Bonitz, R. , Ciarleglio, C. , Knight, J. , Haddad, N. , Klein, K. , Nowak, S. , Klein, D. , Onufer, N. , Glazebrook, K. , Kobeissi, B. , Baez, E. , Sarkissian, F. , Badalian, M. , Abarca, H. , Deen, R. G. , Yen, J. , Myint, S. , Maki, J. , Pourangi, A. , Grinblat, J. , Bone, B. , Warner, N. , Singer, J. , Ervin, J. , & Lin, J. (2018). InSight Mars lander robotics Instrument Deployment System. Space Science Reviews, 214(5), 93 10.1007/s11214-018-0520-7

[jgre21429-bib-0105] Vasavada, A. R. , Piqueux, S. , Lewis, K. W. , Lemmon, M. T. , & Smith, M. D. (2017). Thermophysical properties along Curiosity's traverse in Gale crater, Mars, derived from the REMS ground temperature sensor. Icarus, 284, 372–386. 10.1016/j.icarus.2016.11.035

[jgre21429-bib-0106] Warner, N. H. , Golombek, M. P. , Sweeney, J. , Fergason, R. , Kirk, R. , & Schwartz, C. (2017). Near surface stratigraphy and regolith production in southwestern Elysium Planitia, Mars: Implications for Hesperian‐Amazonian terrains and the InSight lander mission. Space Science Reviews, 211(1‐4), 147–190. 10.1007/s11214-017-0352-x

[jgre21429-bib-0107] Warner, N. H. , Golombek, M. P. , Williams, N. , Hausmann, R. , DeMott, A. & Kopp, M. (2019). Probing the regolith at the InSight landing site using rocky ejecta craters. 50^th^ Lunar and Planetary Science, Abstract #1185.

[jgre21429-bib-0108] Warner, N. H. , Grant, J. A. , Wilson, S. , Golombek, M. P. , DeMott, A. , Charalambous, C. , Hauber, E. , Ansan, V. , Weitz, C. , Pike, T. , Williams, N. , Banks, M. E. , Calef, F. , Baker, M. , Kopp, M. , Deahn, M. , Lethcoe, H. , & Berger, L. (2020). An impact crater origin for the InSight landing site at Homestead hollow: Implications for near surface stratigraphy, surface processes, and erosion rates. Journal of Geophysical Research: Planets, 125, e2019JE006333 10.1029/2019JE006333

[jgre21429-bib-0109] Weitz, C. M. , Grant, J. A. , Warner, N. H. , Golombek, M. P. , Hauber, E. , Ansan, V. Wilson, S. A. , Charalambous, C. , Williams, N. , Calef, F. , Pike, W. T. , Lethcoe‐Wilson, H. , Maki, J. , DeMott, A. , Kopp, M. (2020). Comparison of InSight Homestead hollow to hollows at the Spirit landing site. Journal of Geophysical Research: Planets, 125, e2020JE00643 10.1029/2020JE006435 PMC7507760 32999801

[jgre21429-bib-0110] Williams, N. R. , Golombek, M. P. , Warner, N. H. , Daubar, I. J. , Hausmann, R. B. , Hauber, E. , Ansan, V. , Grant, J. A. , Weitz, C. M. , Wilson, S. , Charalambous, C. , Pike, T. , Lorenz, R. D. , Maki, J. N. , Abarca, H. E. , Ruoff, N. A. , Deen, R. G. , Garvin, J. B. , Parker, T. J. , Calef, F. J. , Lethcoe, H. A. , Berger, L. M. , DeMott, A. , & Kopp, M. (2019). Surface alteration from landing InSight on Mars and its implications for shallow regolith structure. 50^th^ Lunar and Planetary Science, Abstract #2781, Lunar and Planetary Institute, Houston.

[jgre21429-bib-0111] Wilson, S. A. , Warner, N. H. , Grant, J. A. , Golombek, M. P. , DeMott, A. , Kopp, M. , Berger, L. , Weitz, C. M. , Hauber, E. , Ansan, V. , Charalambous, C. , Williams, N. , Calef, F. , Pike, T. , Lethcoe, H. & Hausmann, R. (2019). Crater retention ages at the InSight landing site: Implications for the degradation history of Homestead hollow. 50^th^ Lunar and Planetary Science, Abstract #2162, Lunar and Planetary Institute, Houston.

[jgre21429-bib-0112] Wilson, S. A. , Warner, N. H. , Grant, J. A. , Golombek, M. P. , & Weitz, C. M. (2020). Comparison of crater retention ages at the InSight and Spirit landing sites. 51^st^ Lunar and Planetary Science, Abstract #2247, Lunar and Planetary Institute, Houston.

[jgre21429-bib-0113] Withers, P. , & Smith, M. D. (2006). Atmospheric entry profiles from the Mars Exploration Rovers Spirit and Opportunity. Icarus, 185(1), 133–142. 10.1016/j.icarus.2006.06.013

[jgre21429-bib-0114] Wolff, M. J. , Lopéz‐Valverde, M. , Madeleine, J. B. , Wilson, R. J. , Smith, M. D. , Fouchet, T. , & Delory, G. T. (2017). Radiative process: Techniques and applications In Haberle R. H., Clancy R. T., Forget F., Smith M. D., & Zurek R. W. (Eds.), The atmosphere and climate of Mars (Chap. 6, pp. 106–171). Cambridge, UK: Cambridge University Press 10.1017/9781129060172

[jgre21429-bib-0115] Zurek, R. W. , & Martin, L. J. (1993). Interannual variability of planet‐encircling dust storms on Mars. Journal of Geophysical Research, 98(E2), 3247–3259. 10.1029/92JE02936

[jgre21429-bib-0116] Zurek, R. W. , & Smrekar, S. (2007). An overview of the Mars Reconnaissance Orbiter (MRO) science mission. Journal of Geophysical Research, 112, E05S01 10.1029/2006JE002701

